# Hepatic progenitor cell-originated ductular reaction facilitates liver fibrosis through activation of hedgehog signaling

**DOI:** 10.7150/thno.91572

**Published:** 2024-03-25

**Authors:** Yonghong Hu, Xinyu Bao, Zheng Zhang, Long Chen, Yue Liang, Yan Qu, Qun Zhou, Xiaoxi Zhou, Jing Fang, Zhun Xiao, Yadong Fu, Hailin Yang, Wei Liu, Ying Lv, Hongyan Cao, Gaofeng Chen, Jian Ping, Hua Zhang, Yongping Mu, Chenghai Liu, Chao-Po Lin, Jian Wu, Ping Liu, Jiamei Chen

**Affiliations:** 1 Institute of Liver diseases, Key Laboratory of Liver and Kidney Diseases (Ministry of Education), Shuguang Hospital affiliated to Shanghai University of Traditional Chinese Medicine, Shanghai 201203, China.; 2 Shanghai Key Laboratory of Traditional Chinese Clinical Medicine, Shanghai 201203, China.; 3 Institute of Interdisciplinary Integrative Medicine Research, Shanghai University of Traditional Chinese Medicine, Shanghai 201203, China.; 4 Institute of Surgery of Integrated Traditional Chinese and Western Medicine, Shuguang Hospital affiliated to Shanghai University of Traditional Chinese Medicine, Shanghai 201203, China.; 5 Department of Hepatobiliary Surgery, Shuguang Hospital affiliated to Shanghai University of Traditional Chinese Medicine, Shanghai 201203, China.; 6 Department of Gastroenterology, Shanghai University of Traditional Chinese Medicine Shanghai TCM - Integrated hospital, Shanghai 201203, China.; 7 School of Life Science and Technology, ShanghaiTech University, Shanghai, 201210, China.; 8 Shanghai Clinical Research and Trial Center, Shanghai 201210, China.; 9 Department of Medical Microbiology & Parasitology, MOE/NHC/CAMS Key Laboratory of Medical Molecular Virology, School of Basic Medical Sciences, Fudan University Shanghai Medical College, Shanghai 200032, China.; 10 Department of Gastroenterology & Hepatology, Zhongshan Hospital of Fudan University, Shanghai 200032, China.; 11 Shanghai Institute of Liver Diseases, Fudan University Shanghai Medical College, Shanghai 200032, China.

**Keywords:** liver fibrosis, Gli1, ductular reaction, hepatic progenitor cells, reactive cholangiocytes

## Abstract

**Background:** It is poorly understood what cellular types participate in ductular reaction (DR) and whether DR facilitates recovery from injury or accelerates hepatic fibrosis. The aim of this study is to gain insights into the role of hepatic progenitor cell (HPC)-originated DR during fibrotic progression.

**Methods:** DR in liver specimens of PBC, chronic HBV infection (CHB) or NAFLD, and four rodent fibrotic models by different pathogenic processes was evaluated. Gli1 expression was inhibited in rodent models or cell culture and organoid models by AAV-sh*Gli1* or treating with GANT61.

**Results:** Severity of liver fibrosis was positively correlated with DR extent in patients with PBC, CHB or NAFLD. HPCs were activated, expanded, differentiated into reactive cholangiocytes and constituted “HPC-originated DR”, accompanying with exacerbated fibrosis in rodent models of HPC activation & proliferation (CCl_4_/2-AAF-treated), *Μdr2*^-/-^ spontaneous PSC, BDL-cholestatic fibrosis or WD-fed/CCl_4_-treated NASH-fibrosis. Gli1 expression was significantly increased in enriched pathways *in vivo* and *in vitro*. Enhanced Gli1 expression was identified in KRT19^+^-reactive cholangiocytes. Suppressing Gli1 expression by administration of AAV-sh*Gli1* or GANT61 ameliorated HPC-originated DR and fibrotic extent. KRT19 expression was reduced after GANT61 treatment in sodium butyrate-stimulated WB-F344 cells or organoids or in cells transduced with Gli1 knockdown lentiviral vectors. In contrast, KRT19 expression was elevated after transducing Gli1 overexpression lentiviral vectors in these cells.

**Conclusions:** During various modes of chronic injury, Gli1 acted as an important mediator of HPC activation, expansion, differentiation into reactive cholangiocytes that formed DR, and subsequently provoked hepatic fibrogenesis.

## Introduction

Chronic liver diseases constitute a significant economic, social, and biomedical problem worldwide, with more than 800 million people around the world and about 2 million of them die every year 1. Activation of hepatic stellate cells (HSCs) is considered as a key contributor to liver fibrosis, a major consequence of a variety of chronic liver injury, and hepatocellular injury, inflammatory response, the capillarization of liver sinusoidal endothelial cells also contribute to progression of liver fibrosis 2. Moreover, ductular reaction (DR) is known to be present in most chronic liver diseases; whereas the origin and role of DR are not fully understood, and the underlying mechanisms regulating DR remain to evolve.

DR is pathologically recognized as an increased number of elongated, thin cells that lack easily discernible lumina, and extend irregularly into the lobules, forming a typical bile ductule-like structure 3. Depending on modes of liver injury, ductular reactive cells may be mainly derived from self-proliferating cholangiocytes, differentiation of hepatic progenitor cells (HPCs) or biliary metaplasia of hepatocytes 4. In one study, inhibition of hepatocellular proliferation during chronic injury resulted in significant emergence of cholangiocyte-originated DR. Whereas, in another study, it was revealed that cholangiocytes were initially de-differentiated to HPCs, and then differentiated into functional hepatocytes to facilitate damage repair 5. Subsequently, there was evidence to support for a direct conversion from cholangiocytes to hepatocytes in two models of severe injury without genetic interventions, and the study identified a bi-phenotypic state of cholangiocytes with co-expression of hepatocyte nuclear factor-4α (HNF-4α) and keratin 19 (KRT19) 6. These studies generated growing evidence to believe that DR was beneficial to the repair of liver damage. However, DR was shown to display pro-inflammatory, profibrogenic and pro-angiogenic characteristics to initiate inflammation and fibrosis in alcoholic liver diseases 7, nonalcoholic fatty liver disease (NAFLD) 8, and cholestatic disorders 9. Following apoptosis of DR cells induced by myeloid cell leukemia-1 inhibitor S63845, liver fibrosis was ameliorated 10. Therefore, the role of DR in chronic liver injury is a scientific dilemma with controversial findings in different studies; and in-depth evaluation of DR function in variety of hepatic injury and fibrotic progression is the key step to uncover its pathophysiologic role or attain regenerative recovery.

Glioma-associated oncogene homolog 1 (Gli1) is an effector transcriptional factor of both the canonical and non-canonical hedgehog (Hh) signaling pathways, which are known to regulate embryonic development and tissue repair 11. So far, most studies have stated that aberrant activation of Gli1 in various types of cancers is important in the regulation of cell proliferation, survival, angiogenesis, metabolic reprogramming, and chemotherapeutic resistance 12. It has been reported that canonical Hh/Gli1 signaling regulated HSC-mediated liver angiogenesis promoting liver fibrosis 13. However, it remains largely unknown whether Gli1 regulates HPC-originated DR during fibrotic progression.

In the present study, four rodent models of hepatic fibrosis induced by different pathogenic processes, an *in vitro* model of differentiation of HPCs into cholangiocytes and HPC organoids were used to elucidate the critical role of HPC activation, proliferation and differentiation into reactive cholangiocytes under different modes of insults. DR and fibrosis were determined in various stages of hepatic fibrosis from PBC, CHB and NAFLD patients. The findings underscore that HPC-originated DR occurred in various extent during different modes of hepatic injury, and facilitated the progression of liver fibrosis; and that Gli1 regulated HPC-originated DR, and subsequently accelerated hepatic fibrosis.

## Results

### The degree of liver fibrosis correlated positively with the extent of DR in PBC, HBV-infected and NAFLD patients

**Figure 1:**
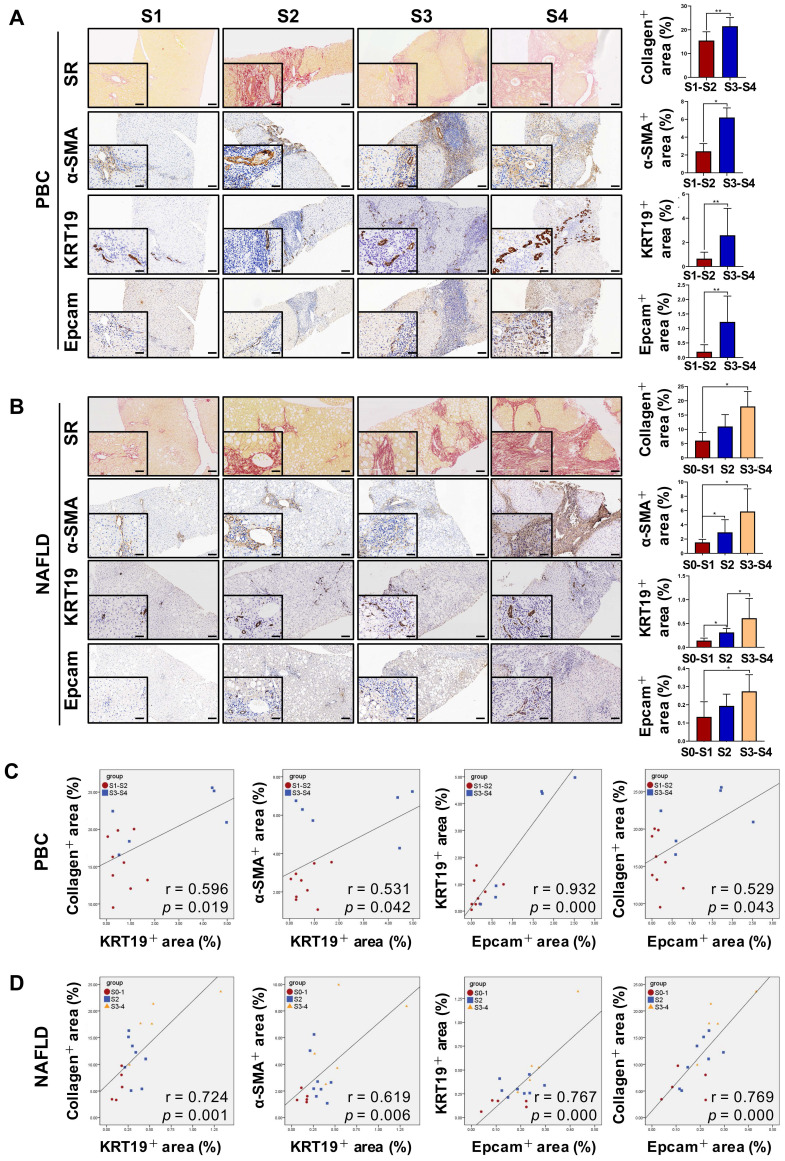
** The correlation among the degree of liver fibrosis, HSC activation and DR in patients with PBC or NAFLD.** (A) Representative images of liver sections stained with SR (scale bar = 200 µm), α-SMA (scale bar = 100 µm), KRT19 (scale bar = 100 µm) and Epcam (scale bar = 100 µm) and morphometric quantification of the SR^+^ area (%), α-SMA^ +^ area (%), KRT19^+^ area (%) and Epcam^+^ area (%) in patients with PBC (*n* = 15) (A) or NAFLD (*n* = 18) (B). The lower left in A and B is the higher magnification (scale bar = 50 µm). Correlation analysis among the collagen^+^ area (%), α-SMA^ +^ area (%), KRT19^+^ area (%) and Epcam^+^ area (%) in patients with PBC (C) or NAFLD (D). Data are represented as mean ± SD (represented by error bars). *, *p* < 0.05; **, *p* < 0.01.* p* values determined by Student's *t* test in (A), one-way ANOVA in (B), and Spearman's correlation analysis in (C), (D).

**Figure 2:**
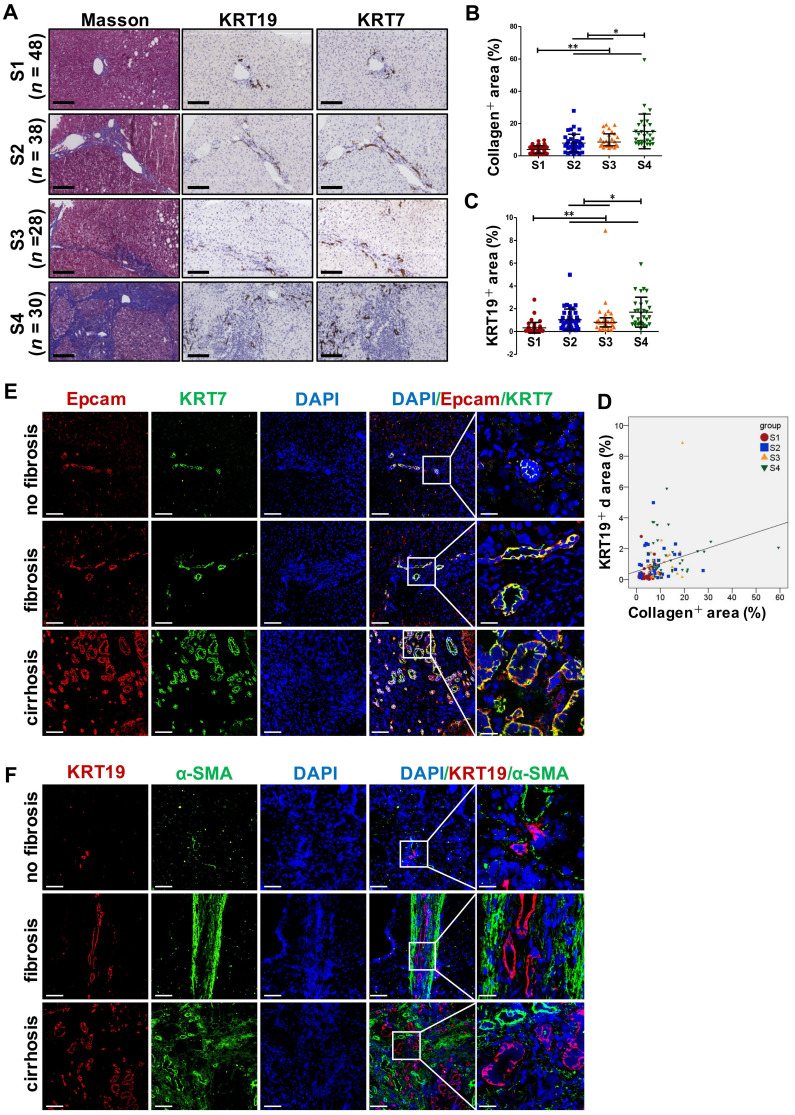
** Collagen expression and immunohistochemical staining in the liver specimens of patients with HBV infection, or hemangioma/paracancerous tissues of patients with hepatic hemangioma and HCC.** A-F showed the results of patients with HBV infection, (A) Representative images of liver serial sections from patients infected with HBV with different stages of liver fibrosis stained with Masson, KRT19 antibody and KRT7 antibody (scale bar = 100 µm) (S1, *n* = 48; S2, *n* = 38; S3, *n* = 28; S4, *n* = 30). (B) Morphometric quantification of the collagen^+^ area stained with Masson (%). (C) Morphometric quantification of the KRT19^+^ area (%). (D) Correlation analysis between the collagen^+^ area (%) and the percentage of KRT19^+^ area (%). E-F showed the results of the hemangioma/paracancerous tissues of patients with hepatic hemangioma and HCC (*n* = 3 per group), (E) Confocal analysis of co-staining for Epcam (red) and KRT7 (green) of frozen sections (scale bar = 100 µm). (F) Confocal analysis of co-staining for α-SMA (green) and KRT19 (red) of frozen sections (scale bar = 100 µm). Nuclei counterstained with DAPI (blue). The right-most column in A and B is the higher magnification of the white box area (scale bar = 25 µm). *, *p* < 0.05; **,* p* < 0.01.* p* values determined by one-way ANOVA in (B), (C), and Spearman's correlation analysis in (D).

DR has been shown to be strongly correlated with disease severity of PBC 14. Therefore, the correlation between the extent of DR, HSC activation and fibrotic degree were initially investigated in liver specimens from PBC patients. As expected, percentage of collagen^+^ or smooth muscle alpha-actin (α-SMA)^ +^ or KRT19^+^ areas with S3-S4 fibrosis in patients was markedly higher than those of S1-S2 patients (*p* < 0.05, *p* < 0.01) (**Figure 1A**). Moreover, both collagen^+^ and α-SMA^+^ areas were positively correlated with the KRT19^+^ area in PBC patients (r = 0.596, *p* = 0.019; r = 0.531, *p* = 0.042) (**Figure 1C, Figure S1A**). Next, the activation status of DR in fibrosis resulting from other chronic liver diseases, such as CHB and NAFLD, was further evaluated. Liver biopsies of 144 CHB patients were classified into 4 categories according to hepatic fibrosis-scoring criteria (S1, *n*=48; S2, *n*=38; S3, *n* = 28; S4, *n* = 30) (**Figure 2A**). The results showed that percentage of collagen^+^ or KRT19^+^ areas with S2, S3, and S4 fibrosis in patients was markedly higher than that of S1 patients (*p* < 0.01). Additionally, the percentage of collagen^+^ or KRT19^+^ area of S4 fibrosis was significantly higher than that of S2 and S3 fibrosis (*p* < 0.05) (**Figure 2B-C**). In accordance, KRT19^+^ area was positively correlated with the collagen^+^ area (r = 0.312, *p* < 0.01) (**Figure 2D**). Consistent with the results in PBC patients and HBV-infected patients, there were positively correlations between both collagen^+^ and KRT19^+^ areas (r = 0.724, *p* = 0.001), as well as α-SMA^+^ and KRT19^+^ areas in NAFLD patients with fibrotic progression (r = 0.619, *p* = 0.006) (**Figure 1B-D, Figure S1B**). In summary, these results demonstrated that the degree of liver fibrosis and HSC activation were positively correlated with the DR extent.

In addition, all paracancerous tissues of surgical patients with hepatic hemangioma and HCC were divided into a non-fibrosis group (*n* = 3), fibrotic group (*n* = 3) and cirrhotic group (*n* = 3) based on SR staining (**Figure S2**). In the fibrotic and cirrhotic groups, KRT19 and KRT7 were expressed in pre-existing bile ducts and reactive cholangiocytes within the fibrous septa. Epithelial cell adhesion molecule (Epcam) is expressed in HPCs and it has been used as a marker to isolate HPCs from human samples 15. In this study, Epcam was expressed in bile duct-like cells in the fibrous septa and occasionally expressed in cells within hepatic lobules (**Figure S2-S3**). Further observations revealed that α-SMA signals encircled KRT19^+^ cells, and KRT19^+^ cells expressed Epcam (**Figure S3**). Through immunofluorescent co-staining of KRT7 with Epcam, KRT19 with α-SMA, it was found that all Epcam^+^ cells expressed KRT7, and KRT19^+^ cells were present in the fibrous septa. Furthermore, with advancement of fibrotic severity, number of Epcam^+^/KRT7^+^ cells was increased significantly (**Figure 2E-F**). In addition, KRT19^+^ area exhibited a significantly positive correlation with the Epcam^+^ area (PBC, r = 0.932, p = 0.000; NAFLD, r = 0.767, p = 0.000) in both PBC and NAFLD specimens (**Figure 1C-D**). Collectively, these findings demonstrated that DR appeared in fibrotic and cirrhotic tissues, and the fibrotic severity correlated with DR extent, and point to that these DR cells might be differentiated from Epcam^+^ HPCs.

### HPCs are activated, proliferated, and mainly differentiated into reactive cholangiocytes, significantly aggravating liver fibrosis

**Figure 3:**
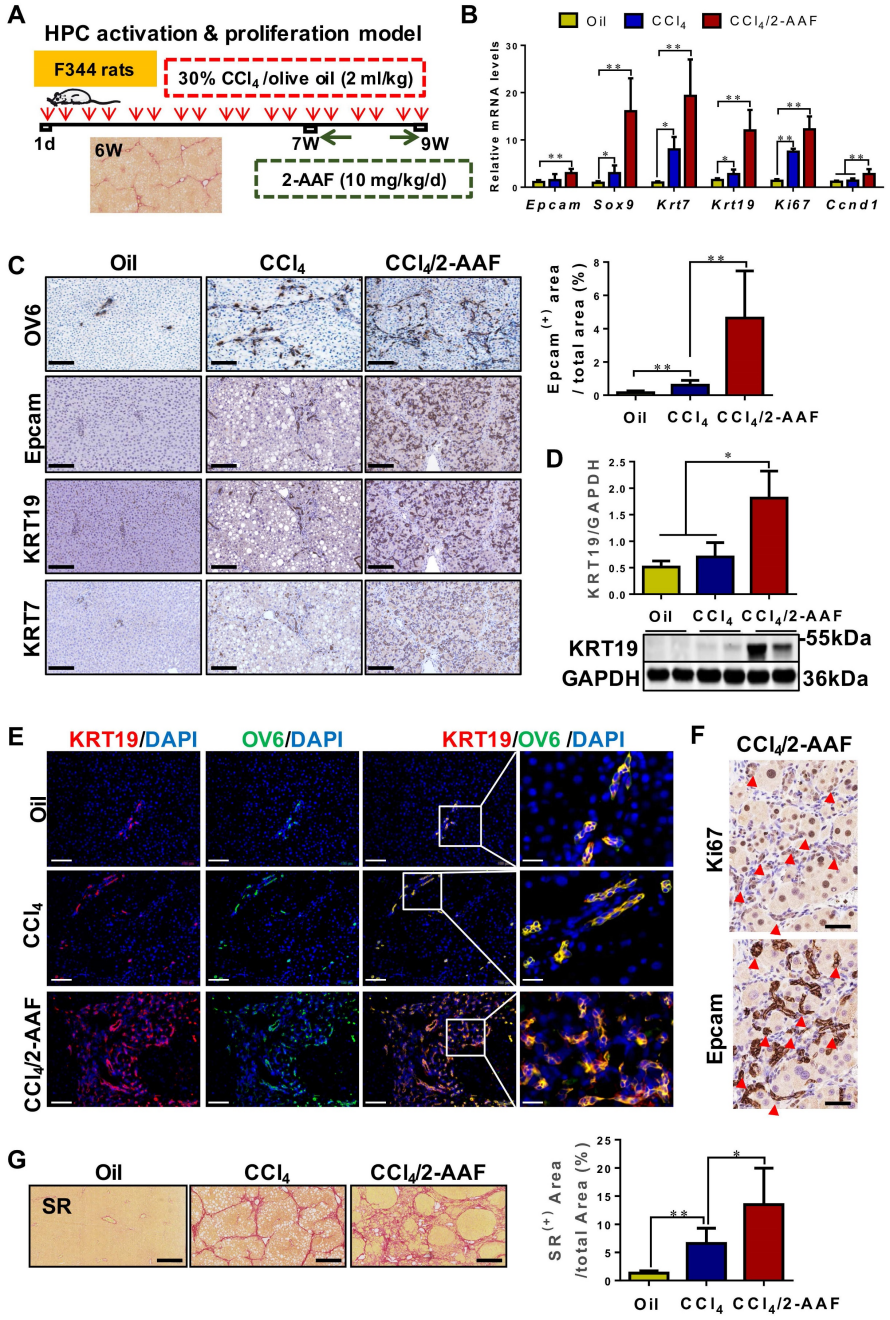
** In CCl_4_/2-AAF-treated rats, 2-AAF induced the activation, proliferation, and differentiation of HPC into cholangiocytes.** (A) Schematic diagram of the experimental design of the HPC activation & proliferation model. (B) Gene expression of *Epcam*, *Sox9*, *Krt7*, *Krt19*, *Ki67*, and *Ccnd1* was determined by qRT-PCR (*n* = 6 per group). (C) Representative staining for OV6, Epcam, KRT19, and KRT7 was performed on paraffin liver sections (scale bar = 100 µm), and morphometric quantification of the Epcam^+^ area (%). (D) Immunoblotting for KRT19. GAPDH was used as loading control (*n*=4 per group). (E) Confocal analysis of co-staining for KRT19 (red), OV6 (green) and DAPI (blue) of frozen sections (scale bar = 100 µm). Higher magnification of the white box area (scale bar = 25 µm) is shown in the right-most column. (F) Representative staining for Ki67 and Epcam in serial sections. The red arrow points to the cells co-expressed Ki67 and Epcam. (G) Representative SR (scale bar = 200 µm) staining and collagen morphometry (%) of SR^+^ area (*n* = 6 per group). *, *p* < 0.05; **,* p* < 0.01. *p* values determined by one-way ANOVA analysis.

HPC activation & proliferation model was established by CCl_4_/2-AAF treatment (**Figure 3A**). The expression of HPC markers: Epcam and Sox9; cholangiocyte markers: Krt7 and Krt19; and cellular proliferation marker: Ki67 and cyclin D1 (Ccnd 1), were markedly higher in the CCl_4_/2-AAF-treated rats than in CCl_4_-treated or controls as determined by qRT-PCR or Western blot analysis (*p* < 0.05, *p* < 0.01) (**Figure 3B-D**). In addition, immunohistochemical staining showed that in the CCl_4_/2-AAF-treated rats, OV6^+^ cells, Epcam^+^ cells, KRT19^+^ cells and KRT7^+^ cells were mainly located within the fibrous septa forming pseudo bile ductule-like structures (**Figure 3C**). Immunohistochemical staining of serial sections showed that Epcam^+^ cells expressed Ki67 in the CCl_4_/2-AAF-treated rats (**Figure 3F**). These findings indicated that HPCs in the CCl_4_/2-AAF-treated rats were in an actively proliferative state, and there were numerous reactive cholangiocytes. To clarify the difference and relationship between HPCs and cholangiocytes, immunofluorescent counter-staining of OV6 with KRT19 was performed. It was evident that almost all OV6^+^ cells expressed KRT19 in the CCl_4_/2-AAF-treated rats (**Figure 3E**), indicating that during fibrotic progression, 2-AAF treatment induced the activation and proliferation of oval cells or so-called HPCs, which predominantly differentiated into reactive cholangiocytes and formed DR under chronic injury with inhibition of hepatocellular proliferation by 2-AFF in rats.

It was further found that OV6^+^ cells were encircled by α-SMA^+^ or Col-I^+^ cells in the CCl_4_/2-AAF-treated rats (**Figure S4**). Additionally, fibrous septa in the CCl_4_/2-AAF-treated rats was thicker than that in the CCl_4_-treated rats, and actually infiltrated into the parenchyma of the lobe and formed the pseudo-lobules, representing typical characteristics of cirrhosis (**Figure 3G**). More convincingly, collagen^+^ areas and the hydroxyproline (Hyp) content (*p* < 0.05) (**Figure 3G, Figure S5B**), as well as the expression of Col-I, Col-IV, α-SMA, and desmin were significantly increased in CCl_4_/2-AAF-treated rats as compared to CCl_4_-treated rats as evidenced by immunohistochemical staining, qRT-PCR and Western blot analysis (*p* < 0.05, *p* < 0.01) (**Figure S5A, S5C-D**). These findings documented that HPC-originated DR was accompanied by worsened liver fibrosis.

To further explore the activation status of DR in liver fibrosis, two mechanistically different biliary fibrosis models, including *Μdr2*^-/-^ spontaneous PSC 16, and BDL-cholestatic fibrosis were investigated. Hepatic expression of Col-I, Col-III, Col*-*IV, α-SMA, TGF-β1, Epcam, KRT19 and KRT7 was significantly increased in the *Μdr2*^-/-^ as compared to WT mice (*p* < 0.01) (**Figure S6**). BDL rats displayed markedly upregulated expression of Col-I, α-SMA, Ccnd1, Ki67, Epcam, Sox9, OV6, KRT19, and KRT7 in the liver as compared to those with sham surgical procedure (*p*<0.01) (**Figure S7**). Moreover, steatohepatitis with fibrosis was successfully established in WD-fed/CCl_4_-treated model as evidenced by H&E and SR staining (**Figure S8A**), hepatic Hyp content (**Figure S8B**), as well as Col-I, Col-III, Col-IV, α-SMA and TGF-β1 expressions (**Figure S8C, S8F**), which were similar to the report 17 and consistent in patients with NASH 8, 18. Furthermore, WD-fed/CCl_4_-treatment significantly upregulated the hepatic expression of Epcam, Sox9, KRT19 and KRT7 (*p* < 0.05; *p* < 0.01) (**Figure S8A, Figure S8D**), and Epcam^+^ cells also expressed KRT7 as indicated by immunofluorescent co-staining (**Figure S8E**). These results suggested there was significant DR and fibrosis in the portal area in this NASH-fibrotic model. Taken together, it is evident that during variety of chronic injuring processes regardless of the cause, DR as a result of HPC activation, expansion, differentiation into reactive cholangiocytes might accelerate hepatic fibrosis.

### Gli1 is involved in HPC-originated DR

**Figure 4:**
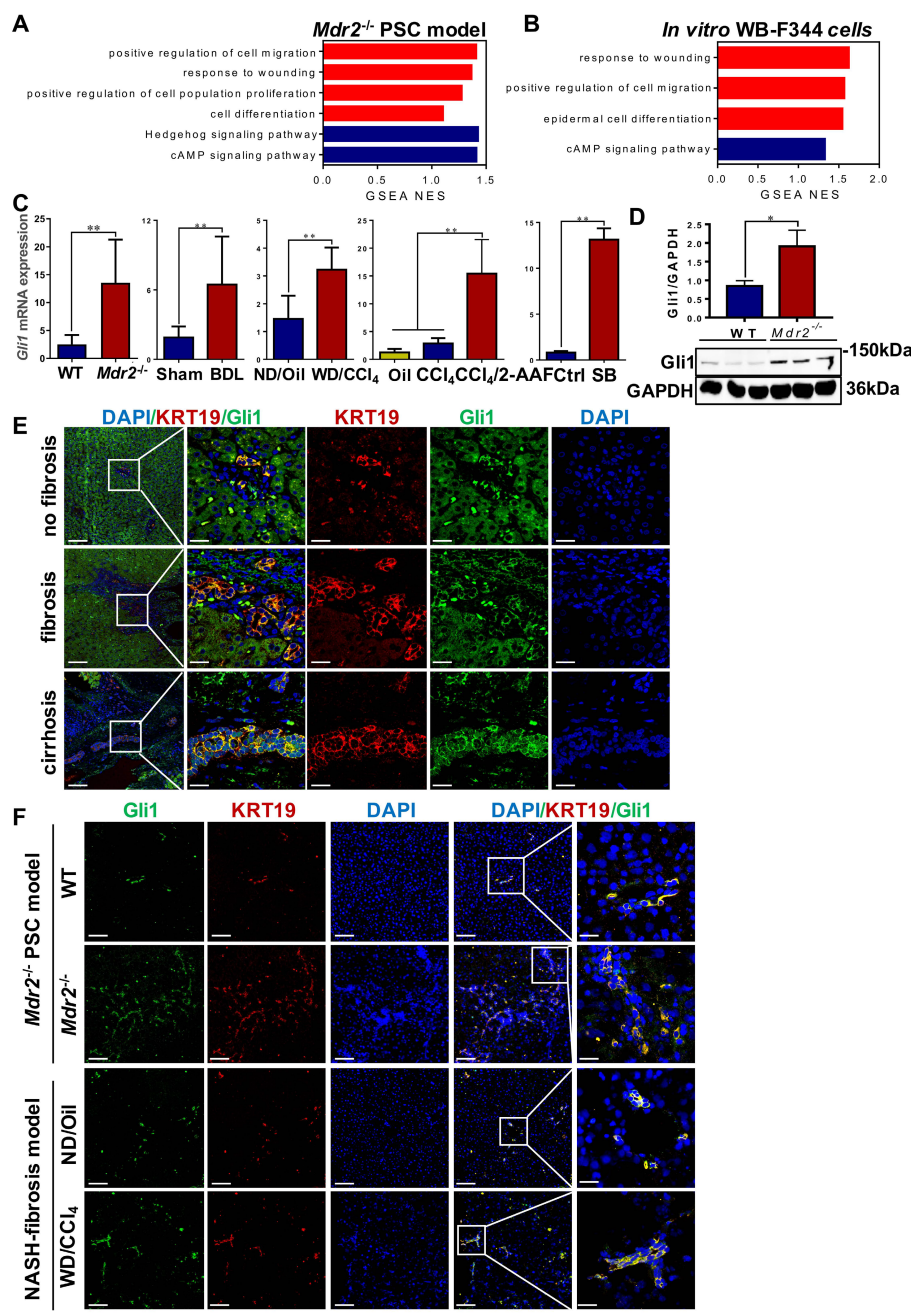
** Gli1 was up-regulated in patients' liver specimens,* in vivo* fibrotic models and *in vitro.*** (A) GSEA of RNA-Seq from WT and *Mdr2*^-/-^ mice liver (*n* = 3 mice/group). (B) GSEA of RNA-Seq from WB-F344 cells treated or not treated with SB (*n* = 3 independent replications). (C) Gene expression of *Gli1* of liver samples in different fibrotic models and WB-F344 cells was determined by qRT-PCR (*n* = 6 per group). (D) Immunoblotting for Gli1 of WT and *Mdr2*^-/-^ mice liver. GAPDH was used as loading control. (E) Confocal analysis of co-staining for KRT19 (red) and Gli1 (green) of paraffin sections in hemangioma/paracancerous tissues of patients with hepatic hemangioma and HCC (scale bar = 100 µm and 25 µm). (F) Confocal analysis of co-staining for KRT19 (red) and Gli1 (green) of paraffin sections in *Mdr2*^-/-^ mice and WD-fed/CCl_4_-treated mice. Nuclei counterstained with DAPI (blue) (scale bar = 100 µm and 25 µm). *, *p* < 0.05; **, *p* < 0.01.* p* values determined by Student's *t* test or one-way ANOVA analysis.

To systematically investigate the mechanisms of HPC activation, expansion, differentiation into cholangiocytes, a transcriptomic assay was performed in *Μdr2*^-/-^ mice and WB-F344 cells, treated with or without sodium butyrate (SB) (**Figure S9A-B**). Through GSEA analyses, it was revealed that the pathways are mostly involved in cell proliferation, migration and differentiation, and response to wounding were activated (**Figure 4A-B**). Heatmaps showed that Gli1 expression was increased in all of these significantly enriched signaling mechanisms (**Figure S10-11**). qRT-PCR and Western blot results further confirmed increased Gli1 expression in four fibrotic models and *in vitro* differentiation of HPCs into cholangiocytes (**Figure 4C-D**). Gli1 was expressed in the KRT19^+^ reactive cholangiocytes in human liver specimens, sections of *Μdr2*^-/-^ mice and WD-fed/CCl_4_-treated mice (**Figure 4E-F, Figure S12**), suggesting that Gli1 appeared to participate in HPC-originated DR.

### Gli1 modulated HPC differentiation into cholangiocyte

**Figure 5:**
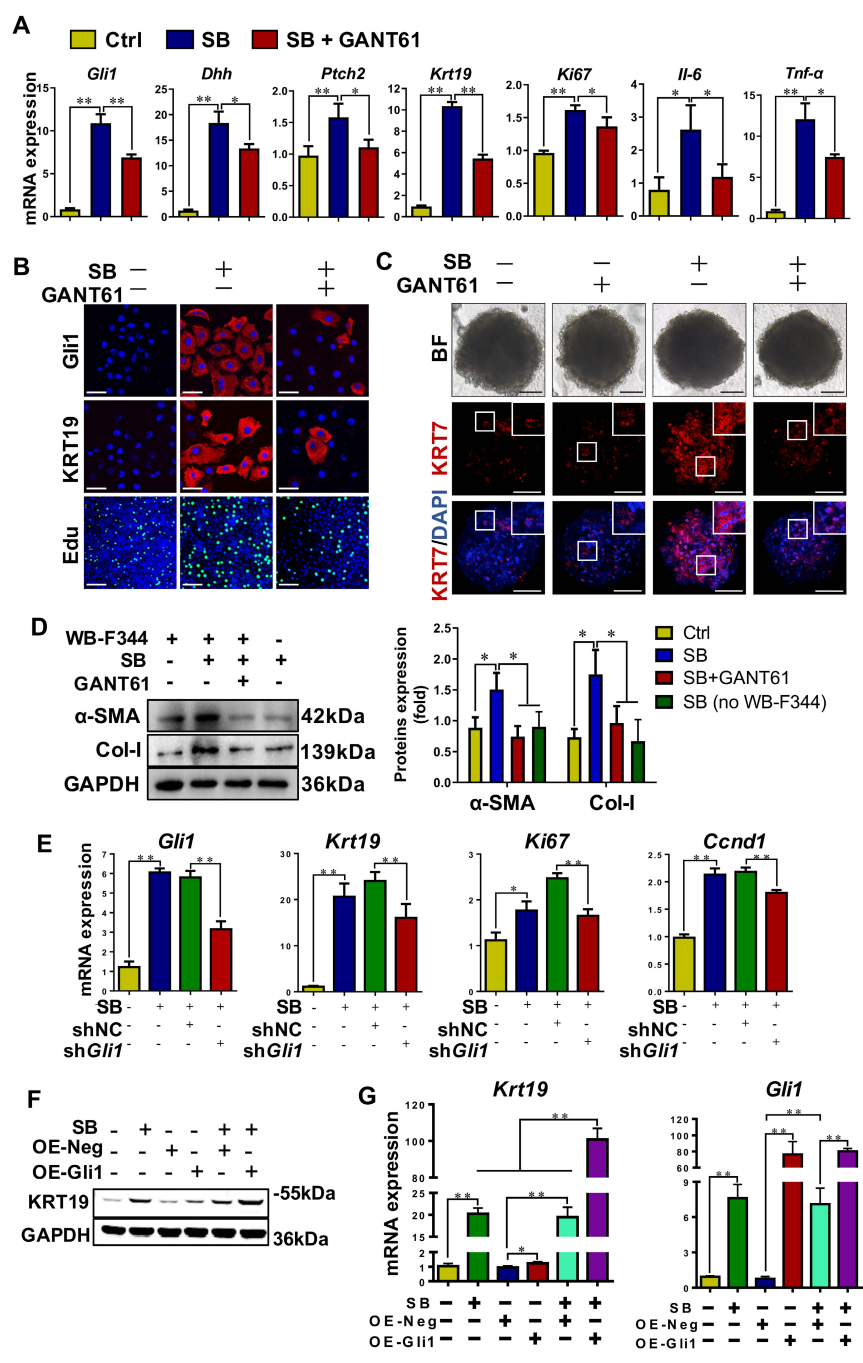
** Gli1 mediated the differentiation of WB-F344 cells into cholangiocytes *in vitro*.** (A) Gene expression of *Gli1*, *Dhh*, *Ptch2*, *Krt19*, *Ki67*,* Il-6* and *Tnf-α* was determined by qRT-PCR. (B) Representative images of WB-F344 cells stained with Gli1, KRT19 (scale bar = 33.3 µm) and Edu (scale bar = 100 µm) with or without GANT61. (C) Representative images of WB-F344 organoids stained with KRT7 (scale bar = 100 µm) with or without GANT61. (D) Immunoblotting and quantification for α-SMA and Col-I production of LX-2 cells in co-culture with WB-F344 cells. GAPDH was used as loading control. (E) Gene expressions of *Gli1*, *Krt19*, *Tnfα*, *Ki67*, and *Ccnd1* after *Gli1* knockdown. (F) Immunoblotting for KRT19 after Gli1 overexpression. GAPDH was used as loading control. (G) Gene expression of* Gli1* and *Krt19* after *Gli1* overexpression. All cell experiments were repeated three times using independent cell cultures. *, *p* < 0.05; **, *p* < 0.01. *p* values determined by one-way ANOVA analysis.

To verify the role of Gli1 on the differentiation of WB-F344 cells into cholangiocytes, SB-induced differentiation of WB-F344 cells was blocked by addition of GANT61, a Gli1 inhibitor. GANT61 at 2 μM was selected for following-up experiments as this concentration did not affect cell viability (**Figure S9C**). The results demonstrated that GANT61 dramatically alleviated SB-induced upregulation of Gli1, KRT19, Dhh, Ptch2, Ki67, interleukin 6 (IL6), and TNF-α (*p* < 0.01; *p* < 0.05) (**Figure 5A-B, Figure S9D**), but abrogated SB-induced increase of Edu^+^ cells (*p* < 0.01) (**Figure 5B, Figure S9E**). WB-F344 cells were co-cultured with human hepatic stellate LX-2 cells to validate the reduction in the differentiation of HPCs into cholangiocytes-activated HSCs by Gli1. The results showed that after LX-2 cells were co-cultured with WB-F344 cells differentiated into cholangiocytes by SB, expression of α-SMA and Col-I in LX-2 cells was significantly upregulated (*p* < 0.05) (**Figure 5D**), suggesting that the differentiation of HPCs into cholangiocytes stimulated HSC activation. Conversely, GANT 61 dramatically inhibited the differentiation of WB-F344 cells into cholangiocytes; subsequently suppressing the activation of HSCs as evidenced by the downregulation of α-SMA and Col-I production in LX-2 cells (*p* < 0.05) (**Figure 5D**). Moreover, to exclude the direct effect of SB on LX-2 cells, we established a transwell subcompartment culture system without WB-F344 cells, the results showed that SB did no exert any influence on the expression of α-SMA and Col-I production in LX-2 cells (**Figure 5D**). In addition, WB-F344 cells were transduced with a lentiviral vector encoding shRNA against* Gli1* for its knockdown (sh*Gli1*). The results showed that after *Gli1* expression was knocked-down in WB-F344 cells (**Figure S9G-H**), mRNA levels of *Krt19, Ki67*, and *Ccnd1* were significantly reduced (*p* < 0.01) (**Figure 5E**). Furthermore, Gli1 overexpression not only directly induced upregulation of KRT19 expression, but also enhanced the SB-induced increased in KRT19 expression (*p* < 0.01) (**Figure 5F-G**).

To further validate the differentiation of WB-F344 cells into cholangiocytes, the formation and differentiation of WB-F344 organoids were conducted. The results showed that WB-344-derived organoids were successfully generated by the 3D organ bud technique with some modifications 19. SB treatment resulted in an increased expression level of the cholangiocyte marker KRT7 compared to the control organoids; whereas, addition of GANT 61 dramatically downregulated the KRT7 expression in organoids (**Figure 5C, Figure S9F**), suggesting that WB-F344 cells were differentiated into cholangiocytes in the format of organoid culture in the presence of SB; on contrast, GANT 61 treatment suppressed their differentiation into cholangiocytes in 3D organoids. In summary, these loss-of-function and gain-of-function experiments verified the notion that Gli1 participated in regulating the differentiation of HPCs into cholangiocytes.

### Inhibition of Gli1 expression repressed DR and ameliorated fibrosis in *Mdr2*^-/-^ PSC mice

**Figure 6:**
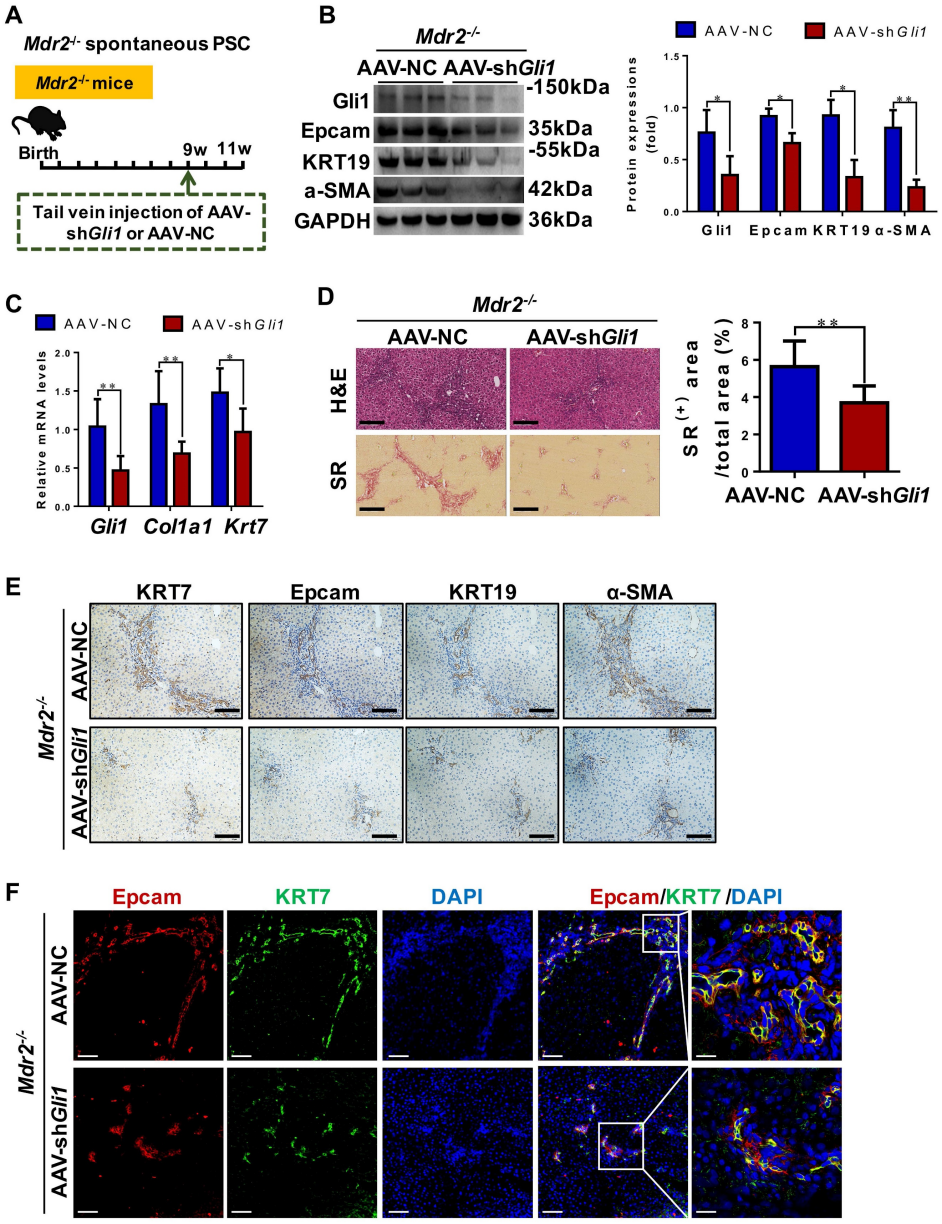
** The degree of DR and hepatic fibrosis in *Mdr2^-/-^* mice after injected with AAV-NC or AAV-sh*Gli1*.** (A) Schematic diagram of the experimental design showed that *Mdr2*^-/-^ mice were injected with AAV-NC or AAV-sh*Gli1* by tail vein at the age of 9 weeks (*n* = 6 per group). (B) Immunoblotting and quantification for Gli1, Epcam, KRT19 and α-SMA. GAPDH was used as loading control (*n* = 3 per group). (C) Gene expressions of *Gli1*, *Col1a1* and *Krt7*. (D) Representative H&E (scale bar = 100 µm), SR (scale bar = 200 µm) staining, and collagen morphometry (%) of SR^+^ area. (E) Representative images of liver sections stained with KRT7, Epcam, KRT19, and α-SMA (Scale bar = 100 µm). (F) Confocal analysis of co-staining for Epcam (red), KRT7 (green) and DAPI (blue) of frozen sections (scale bar = 100 µm). Higher magnification of the white box area (scale bar = 25 µm) is shown in the right-most column. *, *p* < 0.05; **, *p* < 0.01. *p* values determined by Student's *t* test analysis.

To functionally prove the role of Gli1, AAV-sh*Gli1* or AAV-NC vector was injected into *Mdr2*^-/-^ mice through tail vein (**Figure 6A**). Intravenous administration of AAV-sh*Gli1* decreased Gli1 protein and mRNA levels (*p* < 0.05) (**Figure 6B-C**). After AAV-sh*Gli1* intervention, number of KRT19^+^/Gli1^+^ or KRT7^+^/Epcam^+^ cells was significantly declined (**Figure S13, Figure 6F**), mRNA and protein levels of Epcam, KRT19, and KRT7 were significantly decreased (*p* < 0.05) (**Figure 6B-C, 6E**), suggesting that knockdown of Gli1 in mouse liver repressed the DR. Furthermore, treatment with AAV-sh*Gli1* significantly reduced liver fibrosis in *Mdr2*^-/-^ mice, as assessed by inflammatory cell infiltration (H&E staining), collagen (SR staining and the percentage of SR^+^ area), and α-SMA expression (**Figure 6D-E**). These results suggested suppression of Gli1 gene expression by an RNAi approach abrogated HPC expansion and the DR in *Mdr2*^-/-^ mice, and alleviated fibrotic progression. Accordantly, additional data available further confirmed that inhibition of Gli1 expression by treatment with GANT61 repressed HPC-originated DR and fibrosis in *Mdr2*^-/-^ mice (**Figure S14-S15**).

### Inhibition of Gli1 expression repressed the HPC-originated DR, in turn alleviating liver fibrosis in CCl_4_/2-AAF-treated rats

**Figure 7:**
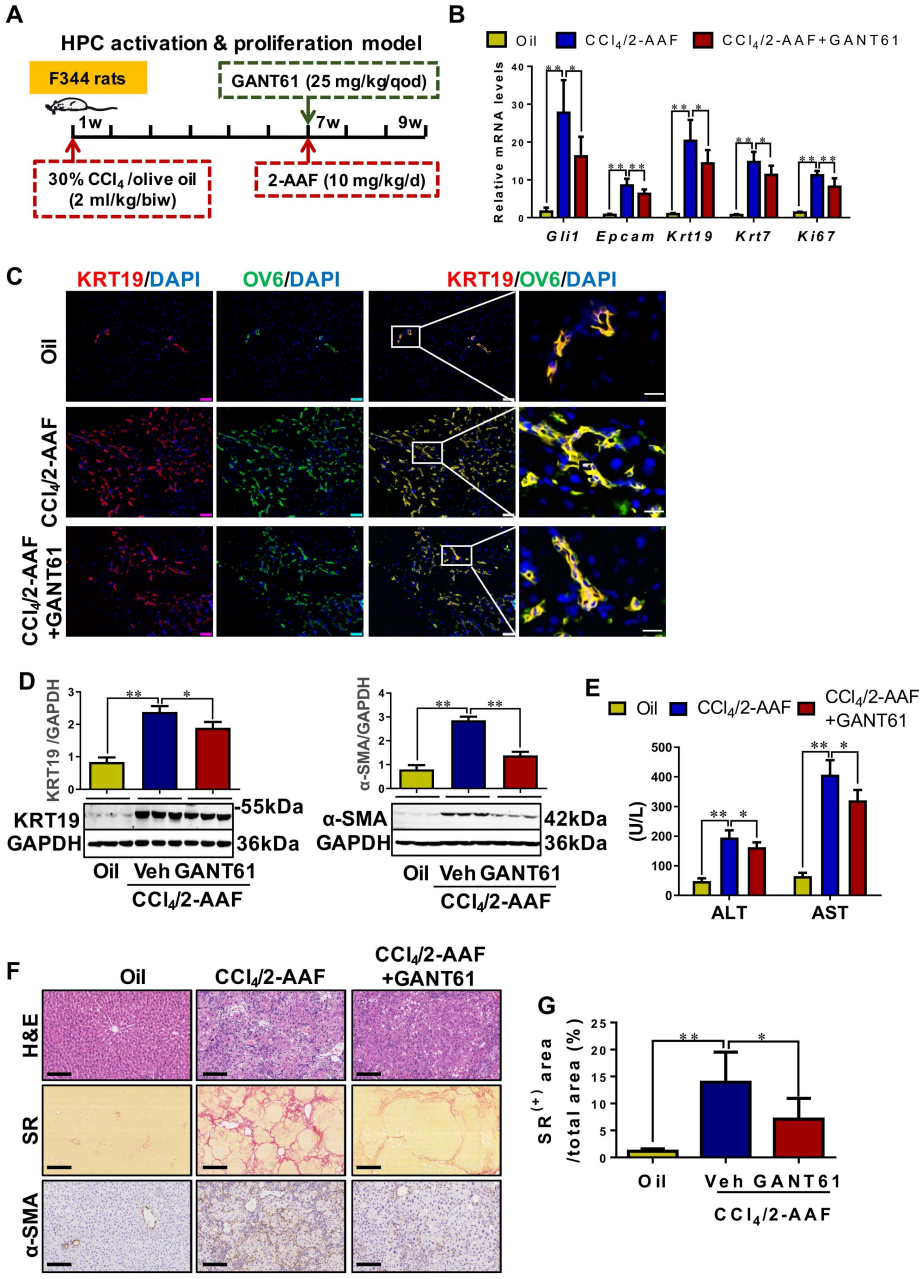
** The effect of GANT61 on DR and liver fibrosis in CCl_4_/2-AAF-treated rats.** (A) Schematic diagram of the experimental design of treatment with GANT61 in CCl_4_/2-AAF-treated rats (*n* = 6-8 per group). (B) Gene expressions of *Gli1*, *Epcam*, *Krt19*, *Krt7*, and *Ki67*. (C) Confocal analysis of co-staining for KRT19 (red) and OV6 (green) of frozen sections (scale bar = 100 µm). The right-most column is the higher magnification of the white box area (scale bar = 25 µm). (D) Immunoblotting for KRT19 and α-SMA. The quantification of KRT19 and α-SMA were measured employing histogram normalized to GAPDH protein based on the results of Western blot (*n* = 3 per group). (E) Serum ALT and AST activities. (F) Representative H&E (scale bar = 100 µm), SR (scale bar = 200 µm), and α-SMA (scale bar = 100 µm) staining. (G) Collagen morphometry (%) of SR^+^ area. *, *p* < 0.05; **, *p* < 0.01. *p* values determined by one-way ANOVA analysis.

To determine the role of Gli1 in the DR of liver fibrosis caused by xenobiotics, rats were treated with CCl_4_/2-AAF plus GANT61 (**Figure 7A**). CCl_4_/2-AAF-cased upregulation of Gli1, Epcam, KRT19, KRT7, and Ki67 was significantly reversed by GANT61 (*p* < 0.01; *p* < 0.05) (**Figure 7B,7D**). Number of OV6^+^, Epcam^+^, KRT19^+^, or KRT7^+^, KRT19^+^/OV6^+^ or KRT7^+^/OV6^+^ cells in the presence of GANT61 was decreased as compared to that of the CCl_4_/2-AAF-treated rats (**Figure S16A-B, Figure 7C**). The administration of GANT61 effectively alleviated hepatic injury, inflammatory cell infiltration, HSC activation and collagen deposition induced by CCl_4_/2-AAF, as indicated by serum ALT and AST activities (*p* < 0.05), H&E and SR staining, as well as comparable levels of α-SMA, Col-I, and Col-IV (*p* < 0.05) (**Figure 7E-G, Figure S16C**). These results indicated that inhibiting Gli1 expression in CCl_4_/2-AAF-treated rats significantly repressed HPC activation, proliferation, and differentiation into reactive cholangiocytes, and meanwhile ameliorated liver fibrosis.

### Inhibition of Gli1 expression repressed HPC-originated DR, in turn attenuating liver fibrosis in rats with BDL

**Figure 8:**
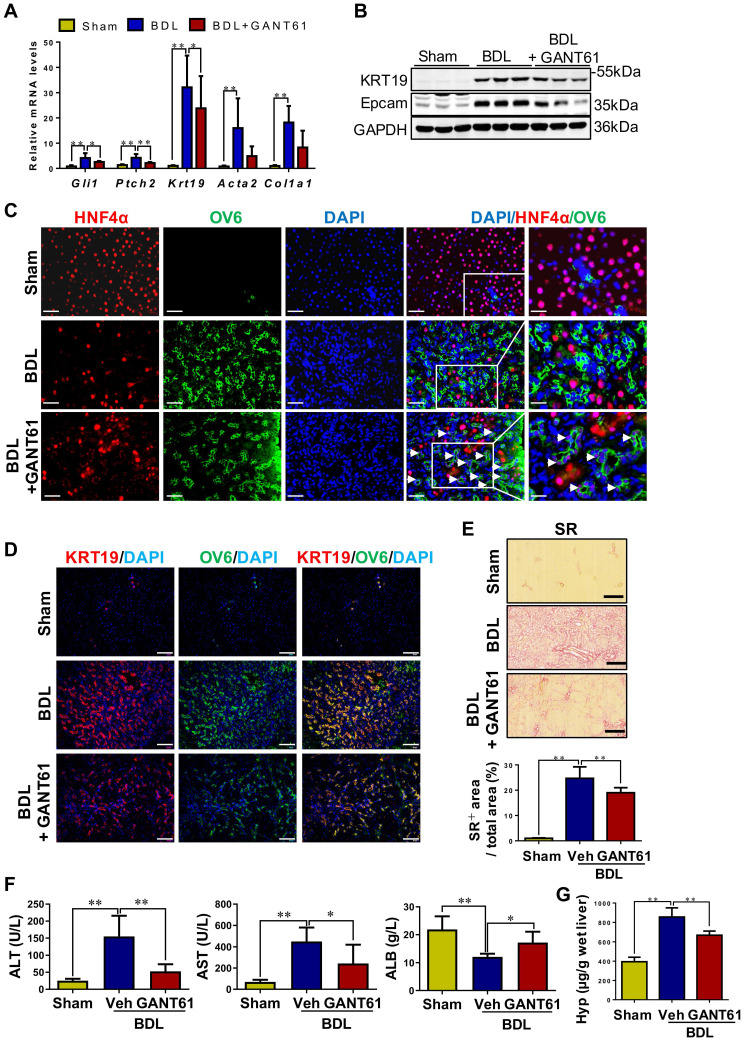
** The effect of GANT61 on DR and the degree of liver fibrosis in BDL rats.** (A) Gene expression of *Gli1*, *Ptch2*, *Krt19*, *Acta2* and *Col1a1* was determined by qRT-PCR. All mRNA values were normalized against *Gapdh* levels and are shown relative to expression level in the control group (*n* = 6-8 per group). (B) Immunoblotting for KRT19 and Epcam. GAPDH was used as loading control (*n* = 3 per group). (C) Confocal analysis of co-staining for HNF4α (red) and OV6 (green) (scale bar = 100 µm). The white arrows indicated the HNF4α^+^/OV6^+^ cells. (D) Confocal analysis of co-staining for KRT19 (red) and OV6 (green) (scale bar = 100 µm). (E) Representative images of liver sections stained with SR (scale bar = 200 µm), and collagen morphometry (%) of SR+ area. (F) Serum ALT and AST activities, and ALB content. (G) Hepatic collagen content as determined biochemically via Hyp. *, *p* < 0.05; **, *p* < 0.01. *p* values determined by one-way ANOVA analysis.

Rats with BDL were treated with GANT61 (**Figure S17A**). In BDL rats, GANT61 markedly reversed the BDL-induced upregulation of Gli1, Ptch2, KRT19, and Epcam (*p* < 0.01; *p* < 0.05) (**Figure 8A-B**). Number of KRT7^+^, or KRT19^+^, or OV6^+^ cells, as well as KRT19^+^/OV6^+^ or KRT7^+^/OV6^+^ cells was significantly decreased in the GANT61-treated rat compared to BDL rats as indicated by immunohistochemistry staining and immunofluorescent co-staining (**Figure 8D, Figure S17C**). Interestingly, there are barely HNF4α^+^/OV6^+^ cells in the liver tissues of Sham or BDL rats, while number of HNF4α^+^/OV6^+^ cells was significantly increased after GANT61 administration (**Figure 8C**). These results suggested that inhibition of Gli1 expression repressed DR in rats undergoing BDL; whereas might promote HPC differentiation into hepatocytes. Further investigation showed GANT61 significantly alleviated BDL-induced accumulation of inflammatory cells (**Figure S17B**), hepatic injury, collagen deposition and HSC activation, as indicated by the reduction in ALT and AST activities (*p* < 0.05, *p* < 0.01) (**Figure 8F**), collagen^+^ areas (**Figure 8E**), Hyp content (**Figure 8F**), as well as α-SMA or Col-I signals encircling OV6^+^ cells (**Figure 8A, Figure S18A-B**), but resulted in an elevation in ALB content (*p* < 0.05) (**Figure 8F**). This observation suggested that inhibition of Gli1 expression suppressed HPC differentiation into reactive cholangiocytes in the rats subjected to BDL; whereas might promote HPC differentiation into functional hepatocytes, which could subsequently alleviate biliary fibrosis.

## Discussion

DR is defined as "a reaction of ductular phenotype, possibly but not necessarily of ductular origin” 3. In fact, the "reaction" encompasses not only the proliferation of ductular reactive cells but also other reactive responses, such as the infiltration of inflammatory cells and the generation of stroma. On the other hand, ductular reactive cells in DR release several cytokines, such as TGF-β1/β2, IL6, TNFα, platelet-derived growth factor, CC-motif chemokine 2, Hh ligands, etc., which subsequently attract infiltration of portal inflammatory cells and enhance activation of portal fibroblasts and HSCs 20, 21. In chronic liver diseases, DR expansion is associated with disease progression and poor patient outcome 22. In both PBC and PSC, DR expansion is associated with more extensive fibrosis, and DR extent strongly correlated with prognostic scores predicting PBC outcome and UDCA-response 23. In line with these studies, liver histopathologic analysis indicated that severity of liver fibrosis in patients with PBC was positively correlated with the DR extent in the present study.

To further demonstrate that DR expansion correlated closely with fibrosis severity, two other liver diseases, CHB and NAFLD were evaluated. Obviously, pathological manifestations of CHB and NAFLD are remarkably different from PBC. The well-documented features of NAFLD, especially the early stages, are the presence of lobular features of injury, including hepatocyte ballooning, Mallory bodies, zone 3 inflammation and perisinusoidal fibrosis, however, portal fibrosis has been identified as a key feature associated with progression of the disease and liver-related mortality 8, 24. Taken together, our observations suggested the extent of DR increased with disease progression and correlated positively with hepatic fibrosis severity, which was a common feature in the progression of liver fibrosis, independently of the cause or etiology.

HPC is a collection of multiple stemness cell populations with spatiotemporal heterogeneity, and differentiates in different directions under a variety of liver injury backgrounds. There is a strong association of HPC activation and DR during fibrosis progression 25. And the appearance of DR is thought to be the result of HPC activation and proliferation 8, 26. High-throughput sequencing verified that DR derived from HPC activation and proliferation could be a driving factor for fibrogenesis and angiogenesis in various modes of chronic liver diseases 27. Epcam is considered as the marker of HPCs and has been used to isolate progenitor cells from human samples 15, 28, 29. OV6 is an acknowledged marker for rodent oval cells 30. In this study, we used Epcam as an HPC marker in human samples and mouse models, and OV6 as an HPC marker in rat models, while KRT19 and KRT7 as the markers of cholangiocytes. Immunohistochemical and immunofluorescent staining showed that α-SMA^+^ cells were surrounded with the KRT19^+^ cells. This evidence documented that there is a close relationship between hepatic fibrogenesis and DR. Further data revealed that many KRT19^+^ or KRT7^+^ ductular reactive cells co-expressed Epcam, suggesting that these ductular reactive cells might be partially originated from HPCs, which was further verified in PBC and NAFLD as evidenced with that α-SMA^+^ area was positively correlated with KRT19^+^ area, and KRT19^+^ area was significantly positively correlated with Epcam^+^ area. Furthermore, various rodent models of hepatic fibrosis by different pathogenic processes provide more solid evidence. Firstly, in the presence of liver fibrosis by sustained xenobiotic (CCl_4_) injury, 2-AAF, as a mitotic inhibitor blocking hepatocellular proliferation 31, activated HPCs, enhanced their proliferation, and subsequent differentiated into reactive cholangiocytes forming a DR worsening liver fibrosis in rats. Furthermore, *Μdr2*^-/-^ spontaneous PSC mice and BDL cholestatic rats, which represented two mechanistically different biliary fibrosis models; and a non-biliary fibrosis model, WD-fed/CCl_4_-treated NASH-fibrosis mice, were established to mimic histological, immunological and transcriptomic features of human NASH, led to steatohepatitis and extensive fibrosis 17. It has been demonstrated that activation and proliferation of HPCs and the DR generation were confirmed in various extent in these patients and rodent models, and they were found to remarkably accompany with exacerbated liver fibrosis.

Gli proteins comprise a relatively large family of multifunctional transcription factors, which belong to C2H2 zinc-finger proteins, and play a pivotal role in regenerative and repairing processes. Gli1 is not only a transcriptional activator, but also a target gene of Gli proteins 32, thereby amplifying the transcriptional responses of Hh signaling. In healthy adult livers, Hh is in a quiescent state. However, when injury occurs, Hh signaling pathway is activated and plays an essential role in coordinating hepatic repair. However, when injury persists, Hh signaling pathway controls HSC activation 33 or promotes sinusoidal endothelial cell capillarization 34 to correspondingly advance fibrotic progression. An investigation demonstrated that Hh ligands were mainly produced by cholangiocytes and HPCs in PBC and PSC patients, and cholangiocytes and HPCs responded to Hh ligands, which consequently activated Hh signaling pathway in these cells under biliary insults 35. Animal experiments demonstrated that as Hh effector cells, HPCs were under control for their proliferation, apoptosis, migration, and differentiation by Hh signaling pathway 36. In the present study, Gli1 overexpression was confirmed in four different fibrotic models and *in vitro* experiments. Further immunofluorescent staining was performed in human liver specimens and liver sections of the *Mdr2*^-/-^ mice and WD-fed/CCl_4_-treated mice, and it was evident that Gli1 was mainly expressed in the KRT19^+^ cells, suggesting that reactive cholangiocytes might be originated from HPCs, and are probably Gli1 responding cell types during DR and fibrotic progression.

To verify the role of Gli1 in the processes of HPC-originated DR, the Gli1 inhibitor GANT61 was used to treat SB-stimulated WB-F344 cells, an *in vitro* model of differentiation into cholangiocytes of HPCs, or WB-F344 organoids. Inhibition of Gli1 by GANT61 or shRNA resulted in reduced expression of KRT19 and Ki67, as well as proinflammatory cytokines including IL-6 and TNF-α in the SB-stimulated cells. Further experiments confirmed that KRT19 expression was significantly elevated after transducing WB-F344 cells with a Gli1 overexpression lentiviral vector. Interestingly, Smo, a key transporter of the canonical Hh pathway, was not activated* in vitro*, suggesting that non-canonical Hh/Gli1 signaling pathway may play a key role in HPC-originated DR. These results suggested that Gli1-mediated activation and expansion of HPCs, and facilitated their differentiation into cholangiocytes.

Moreover, after treating *Μdr2*^-/-^ PSC mice with AAV-sh*Gli1*, or *Μdr2*^-/-^ mice, CCl_4_/2-AAF-treated rats, and BDL rats with GANT61, HPC proliferation and DR extent were significantly alleviated, suggesting that inhibition of Gli1 expression could repress HPC-originated DR during hepatic fibrogenesis although it could not exclude the role of GANT61 on other cell types in the liver. HPCs are facultative stem cells that reside in the portal zone and may differentiate towards hepatocellular or cholangiocytic lineages 37. For the first time, this study demonstrated that inhibition of Gli1 expression suppressed DR in rats with BDL; whereas promoted HPC differentiation into functional hepatocytes, which subsequently alleviated biliary liver fibrosis. Therefore, it appears that full recuperation of liver-specific functions requires precise modulation of Hh signaling since complete differentiation of HPCs into mature liver epithelial cells seems to require repression of Hh signaling activity.

It is known that Gli1 is closely associated with the formation and development of fibrosis in various organs, such as liver, kidneys and lungs 38. And similar to HPCs, HSCs are also Hh-responsive. And Hh signaling drives the trans-differentiation of quiescent HSCs into myofibroblasts by direct preferential induction of a metabolic process (glycolysis) 33 and regulating glutaminolysis 39. There exists evidence that inhibiting the Hh pathway, either pharmacologically 40 or through conditional disruption of *Smo* gene 41 reverses liver fibrosis in BDL mice. With intervention with AAV-sh*Gli1* or GANT61, inflammatory cell accumulation, HSC activation and collagen deposition were considerably reduced in *Μdr2*^-/-^ mice, CCl_4_/2-AAF-treated rats or BDL rats in the present study. These data demonstrated that the suppression of HPC-originated DR by inhibition of Gli1 attenuated hepatic fibrosis, since ductular reactive cells may stimulate HSC/myofibroblast activation through secretion of multiple proinflammatory and profibrogenic factors, which was evidenced by co-culture of WB-F344 cells with LX-2 cells. In consistency, in patients with PBC or NAFLD, there was positive correlation between α-SMA^+^ area and KRT19^+^ area in this study. Taken together, various rodent models of hepatic fibrosis demonstrated that inhibition of Gli1 mitigated liver fibrosis by at least two distinct and independent mechanisms: 1) suppressing HPC-originated DR but promoting HPC differentiation into functional hepatocytes; and 2) inhibiting HSC activation.

In conclusion, during the progression of liver fibrosis, HPC-originated DR worsened hepatic fibrosis regardless of the cause or etiologies. Gli1, a transcription factor of hedgehog signaling, acted as an important mediator of HPC activation, expansion, differentiation into reactive cholangiocytes that formed DR, and subsequently accelerated hepatic fibrogenesis. The findings of the present study support that Gli1 may serve as a potential therapeutic target to ameliorate liver fibrosis caused by various modes of chronic insults.

## Materials and Methods

### Clinical liver biopsy specimens

All experiments on clinical specimens were informed consent retrieved from patients and approved by the Institutional Review Board at Shuguang Hospital Affiliated to Shanghai University of Traditional Chinese Medicine, Shanghai, China (NO. 2015-445-73-02) conformed to the ethical guidelines of the 2013 Declaration of Helsinki as reflected in a prior approval by the institution's human research committee. Liver biopsy specimens of 15 patients with PBC, 144 patients with hepatitis B virus (HBV) infection, and 18 patients with NAFLD were collected from Shuguang Hospital Affiliated to Shanghai University of Traditional Chinese Medicine, Shanghai, China, and Xiamen Hospital of Traditional Chinese Medicine, Xiamen, China. Hemangioma/paracancerous liver tissues of surgical patients with hepatic hemangioma and hepatocellular carcinoma (HCC) were collected from Shuguang Hospital Affiliated to Shanghai University of Traditional Chinese Medicine.

### Preparation of animal models

HPC activation & proliferation model (CCl_4_/2-AAF-treated), *Mdr2*^-/-^ spontaneous PSC mice, BDL-cholestatic fibrosis in rats and WD-fed/CCl_4_-treated NASH-fibrosis model were established. All animal experiments were performed in accordance with national guidelines and approved by the Experimental Animal Ethics Committee of Shanghai University of Traditional Chinese Medicine, Shanghai, China (Shanghai University of Traditional Chinese Medicine Animal Experiment Guide) (NO. 201608001, NO. PZSHUTCM190315012, NO. PZSHUTCM200731007, and NO. PZSHUTCM190719002).

### Cell culture and SB-induced WB-F344 cell differentiation

Hepatic progenitor cell line WB-F344 was cultured for 4 days in DMEM medium containing 10% FBS and SB (Sigma-Aldrich, B5887) at a final concentration of 3.75 μM to differentiate into cholangiocytes. Control cells were cultured in medium free of SB. To inhibit Gli1 expression, WB-F344 cells were treated with GANT61 or transduced with lentiviral vectors encoding shRNA against* Gli1*. In contrast, transducing Gli1 overexpression lentiviral vectors in WB-F344 cells to elevate Gli1 expression. All cell experiments were repeated three times using independent cell cultures.

### The formation and differentiation of WB-F344 organoids

WB-344 organoids were generated by the 3D organ bud technique with some modifications 19. For each organoid, 270,000 WB-F344 cells and 3,000 human mesenchymal stem cells (MSCs) were mixed, centrifuged at 200×g for 5 min, resuspended in 200 μL of complete medium (DMEM/F12 medium supplemented with 10% FBS, 2 mM glutamine and 1% penicillin/streptomycin), and seeded onto one well of 96-well Clear Round Bottom Ultra-Low Attachment Microplate (Corning, 7007). The plate was then centrifuged at 300×g for 3 min to gather the cells. After incubating at 37°C for 24 hours, dense, round cell aggregates were observed. For treatment, 100 mL of the culture medium in each well was replaced by same volume of fresh complete medium containing 7.5 mM sodium butyrate and/or 4 mM GANT61 (2× concentration). The medium was replaced daily and organoids were collected after four days of culture for immunofluorescence staining.

### Statistics

Statistical analyses were performed for all results via the SPSS21.0 software package. All measurement data were presented as the mean ± SD unless otherwise specified. Differences between two groups were assessed using Student's *t* test. One-way analysis of variance (ANOVA) followed by the least significant difference test was performed for multiple comparisons between groups. The Pearson correlation coefficient was used. *p* < 0.05 was considered statistically significant.

The detailed Materials and Methods were provided in the Supporting Information.

## Supplementary Material

Supplementary materials and methods, figures, tables.

## Abbreviations

AAV: adeno-associated virus
2-AAF: 2-acetylaminofluorene
BDL: bile duct ligation
CCl_4_: carbon tetrachloride
Col-I: collagen type I
DDC: 3,5-diethoxycarbonyl-1,4-dihydrocollidine
DR: ductular reaction
ECM: extracellular matrix
Epcam: epithelial cell adhesion molecule
Gli1: Glioma-associated oncogene homolog 1
GSEA: gene set enrichment analysis
HBV: hepatitis B virus
HCC: hepatocellular carcinoma
Hh: Hedgehog
HNF-4α: hepatocyte nuclear factor-4α
HPCs: hepatic progenitor cells
Hyp: hepatic hydroxyproline
HSCs: hepatic stellate cells
H&E: hematoxylin-eosin
KRT19: keratin 19
Mdr2: multidrug resistant 2
NAFLD: non-alcoholic fatty liver disease
NASH: non-alcoholic steatohepatitis
PBC: primary biliary cholangitis
PSC: primary sclerosing cholangitis
qRT-PCR: quantitative reverse transcript polymerase chain reaction
Smo: Smoothened
SR: sirius red
SB: sodium butyrate
TGF: transforming growth factor
α-SMA: smooth muscle alpha-actin
WD: Western diet
