# Supplementary material for: Hepatic progenitor cell-originated ductular reaction facilitates liver fibrosis through activation of hedgehog signaling
Source: Theranostics. 2024 Mar 25;14(6):2379–95. doi: 10.7150/thno.91572 (PMC11024850; doi:10.7150/thno.91572)
Supplement: Supplementary file 1 — Supplementary materials and methods, figures, tables. [file thnov14p2379s1.pdf]

Supplementary material

Materials and Methods

Clinical liver biopsy specimens and assessment of liver histology

Liver biopsy specimens of 15 patients with PBC, 144 patients with HBV infection, and 18 patients with NASH were fixed by formaldehyde, dehydrated, embedded in paraffin, and 4-μm thick pathological sections were prepared. Masson staining, SR staining, as well as KRT19, KRT7, Epcam and α-SMA immunohistochemical staining, was subsequently performed. Staging of hepatic fibrosis in all liver biopsy specimens was evaluated through a blinded method by two pathologists according to the Scheuer scoring system[1]. Liver biopsy specimens of patients were classified into 4 categories according to hepatic fibrosis-scoring criteria (PBC: S1, *n* = 2; S2, *n* = 7; S3, *n* = 1; S4, *n* = 5; CHB: S1, *n* = 48; S2, *n* = 38; S3, *n* = 28; S4, *n* = 30; NAFLD: S0-1, *n* = 5; S2, *n* = 8; S3, *n* = 4; S4, *n* = 1). Whole-tissue scanning was performed for all sections via an automatic slide scanner (Model SCN400, Leica Biosystems, Shanghai, China), and photometric analysis for the positive staining (collagen, KRT19, Epcam and α-SMA) was performed by software (APerio ImageScoPe, v12.3.2.5030) and calculated based on whole section area for these sections.

Hemangioma/paracancerous liver tissues of surgical patients with hepatic hemangioma and HCC were fixed in 10% neutral-buffered formalin, dehydrated, and embedded in paraffin for serial sections or embedded in optimal cutting temperature compound for cryosections. Paraffin serial sections were used for H&E and SR staining, as well as immunohistochemical staining for α-SMA, KRT19, Epcam, and KRT7. Whole-tissue section scanning was performed for these sections via an automatic slide scanner. All specimens were divided into a non-fibrosis group (*n* = 3) from hemangioma liver tissues, liver fibrosis group (*n* = 3), and cirrhosis group (*n* = 3)

from paracancerous liver tissues based on SR staining. Immunofluorescent co-staining of KRT19 and Gli1 was performed with paraffin sections, and immunofluorescent co-staining of Epcam and KRT7, as well as KRT19 and α-SMA, was performed in cryosections.

Small molecule inhibitor

The Gli1 inhibitor GANT61 (S8075) was purchased from Selleck Chemicals, Shanghai, China.

Preparation of animal models and drug administration

Female Fisher 344 rats (6-8 weeks old; 160-180 g) and male Sprague Dawley (SD) rats (6-8 weeks old; 160-180 g) were purchased from Vital River Laboratory Animal Technology (Beijing, China). *Mdr2*$^{-/-}$ and *Mdr2*$^{+/+}$ wild-type mice were purchased from Shanghai Research Center of the Southern Model Organisms (Shanghai, China). C57BL/J mice (20-22 g) were purchased from SLAC Laboratory (Shanghai, China).

All experimental animals were housed or bred, and maintained under specific pathogen-free (SPF) conditions in the Animal Experiment Center of Shanghai University of Traditional Chinese Medicine (Shanghai, China), with constant temperature and humidity and a 12-hour light/dark cycle. Animals were fed standard chow and had access to water *ad libitum*.

HPC activation & proliferation model (CCl_4/2-AAF-treated): Fisher 344 rats were injected subcutaneously with a 30% CCl_4 in olive oil solution at a dose of 2 mL/kg of body weight twice weekly for 6 weeks in order to induce liver fibrosis. At the beginning of 7th week, model rats were randomly divided into the CCl_4-treated group (CCl_4-treated group), the CCl_4 combined with 2-AAF (2-AAF dissolved in PEG400, 10 mg/kg/d)-treated group (CCl_4/2-AAF-treated group), the 2-AAF/CCl_4 plus GANT61-treated group (GANT61, 25 mg/kg of body weight; gavage, once every other day) [2].

Rats were gavaged with 2-AAF and simultaneously injected subcutaneously with 30% CCl_4 in olive oil solution in order to promote massive proliferation of HPCs during continuous development of hepatic fibrogenesis. Another group of rats received an equal amount of subcutaneous olive oil injection and equal amount of normal saline by gavage as control (Oil group). Rats were euthanized under sodium pentobarbital i.p. injection at the end of 9th week, and liver tissues were collected for analysis (**Fig. 3A**).

***Mdr2$^{-/-}$* spontaneous PSC mice:** Due to a deficiency of canalicular phospholipid translocase in *Mdr2$^{-/-}$* mice, the lack of phospholipids in the bile results in spontaneous liver injury, and consequently progresses to primary sclerosing cholangitis [3]. *Mdr2$^{-/-}$* mice were randomly divided into the control adeno-associated virus vector (AAV-NC) group and AAV-sh*Gli1* group, or the solvent-treated group and the GANT61-treated group (GANT61, 50 mg/kg of body weight; gavage, once every other day) at 8 weeks of age. Mice were all euthanized at the end of the 11th week, and liver tissues were collected for analysis.

BDL-cholestatic fibrosis in rats: SD rats were randomly divided into the sham-operated group (Sham group) and the BDL group. All rats were anesthetized via inhalation of 5% isoflurane, and anesthesia was maintained with 1.5% isoflurane. Abdominal fur was shaved, and the shaved abdominal skin was sterilized in rats. The abdomen was opened with a midline laparotomy, and the common bile duct, left hepatic duct, and right hepatic duct were separated. The abdomen was then closed for the Sham group. For the BDL group, the left and the right hepatic ducts were ligated. Additionally, the upper end of the common bile duct near the confluence of hepatic ducts was ligated once and the lower end near the duodenum was ligated, and then the abdomen was closed. One week post BDL establishment, rats were randomly

divided into the GANT61-treated group (GANT61, 25 mg/kg of body weight; gavage, once every other day) and the model group. The rats were euthanized at the end of the fourth week, and liver tissues were collected for analysis.

WD-fed/CCl_4-treated NASH-fibrosis model: C57BL/J mice were randomly divided into a normal control group and a WD-fed/CCl_4-treated group. WD-fed/CCl_4-treated mice were given high-fat, high-fructose, and high-cholesterol diet as previously described [4], which contained 21.1% fat, 41% sucrose, and 1.25% cholesterol (Teklad diets, TD, 120528), in addition to a high sugar solution containing 23.1 g/L d-fructose (Sigma-Aldrich, F0127, Shanghai, China) and 18.9 g/L d-glucose (Sigma-Aldrich, G8270). Diet administration and subcutaneous injections of 10% CCl_4 in olive oil solution (0.02 mL/10 g of body weight; once per week) started simultaneously. Mice were euthanized at the end of 16th week, and liver tissues were collected for analysis.

Adeno-associated virus 8 (AAV8) transduction: AAV-NC or AAV-sh*Gli1* vectors were injected with 100 μL of virus containing 2.5 × 10^{11} AAV8 vector genome copies/mouse via the tail vein in *Mdr2*$^{-/-}$ mice. The sh*Gli1* was cloned into an AAV8 package vector pAAV-U6-shRNA/spgRNA v2.0-CBh-EGFP-WPRE. The sequence of the sh*Gli1* was as follows (5'-3'): CCTGTGTACCACATGACTCTA. AAV-NC and AAV-sh*Gli1* vectors were packaged and purified by Obio Technology (Shanghai, China).

Cell culture and SB-induced WB-F344 cell differentiation

Hepatic progenitor cell line WB-F344 was purchased from Xiangf Bio (Shanghai, China). Cells were grown in DMEM high glucose medium (Thermo Fisher, 11965092) containing 10% fetal bovine serum (FBS) (Thermo Fisher, 10100154) and cultured at 37°C with 5% CO_2 concentration and 95% relative humidity in an incubator. Once cells were in 80-90% confluence, they were passaged, cryopreserved, or inoculated to multiple-well plates for experiments.

Co-culture system of WB-F344 cells and HSCs

Transwell (0.4 μm, Corning, USA) was used for co-culture of WB-F344 cells with LX2 cells, a human HSC line. WB-F344 cells were cultured in the lower chamber of the transwell, and SB was used to induce WB-F344 cell dedifferentiation. After two days of treatment with SB, LX-2 cells, which was pre-cultured in upper chamber of transwell for 24 h, ,were co-cultured in upper chamber with WB-F344 cells in lower chamber for another two days in transwells and were collected, separately. All cell experiments were repeated three times using independent cell cultures.

***Gli1* RNAi lentivirus and overexpressing lentivirus vectors**

Gli1 gene RNAi lentivirus vector and empty control lentivirus vector were prepared by Shanghai Genechem Co., LTD (Shanghai, China). The lentivirus vectors carried a green fluorescent label (Vector name, GV248; component sequence, hU6-MCS-Ubiquitin-EGFP-IRES-puromycin; control serial number, CON077; control inserted sequence: 5' TTCTCCGAACGTGTCACGT 3'. RNAi target inserted sequence: 5' CAGCCCTGTGTTCCACATGAT 3'). The *Gli1* gene overexpressing lentivirus vector also carried a green fluorescent label (Vector name, GV367; component sequence, Ubi-MCS-SV40-EGFP-IRES-puromycin; control serial number, CON238; AgeI/NheI enzymatic digestion).

Edu staining

The Cell-LightTM Edu Apollo®488 In Vitro Imaging Kit (Ribobio, Guangzhou, China) was used to assess cell proliferation. Briefly, cells were subjected to Edu labeling for 2 hours following the manufacturer's protocol. Visualization of Edu incorporation was performed using the ArrayScan VTI HCS Reader and quantitation was carried out using Cell Health Profiling BioApplication Software.

Hydroxyproline (Hyp) content measurement

Liver tissues (100 mg) were measured for Hyp content based on the improved method by Jamall *et al* [5]. In brief, liver tissue homogenates were hydrolyzed in hydrolyzing solution at 110°C for 18 hours. After the homogenate was filtered through filter paper, chloramine-T was added to the hydrolysate, and mixed. Then dimethylaminobenzaldehyde at 410 mM (Sinopharm, Shanghai, China) was added and the solution was mixed, followed by incubation at 60°C for 30 min. The OD value at 560 nm was recorded after the samples cooled to room temperature. As an indirect measurement of tissue collagen content, hepatic Hyp content was presented as wet weight (mg/g). The Hyp standard was purchased from Sigma-Aldrich.

Histopathology and immunohistochemical analysis

Liver sections were embedded in paraffin, and were cut at a thickness of about 4 μm. Sections were stained with H&E (lot. 20161225, NJBI, Nanjing, China) and SR to evaluate liver injury and fibrosis status, respectively. As described previously, immunohistochemical staining was performed using paraffin-embedded liver sections or 8-μm thick OCT-embedded frozen liver sections. Briefly, sections were deparaffinized and washed, and the antigens were retrieved, and endogenous peroxidase activity was blocked. Sections were then blocked with 10% goat serum and incubated with primary antibodies overnight at 4°C. After washing, sections were incubated with the corresponding secondary antibodies conjugated with HRP (GTVision III Immunohistochemical Detection Kit, HRP/DAB, anti-mouse/rabbit IgG, two-step, GK5005/5007, Gene Tech, Shanghai, China). The slides were then washed, colored with DAB, counterstained with hematoxylin, washed, dehydrated and sealed. Stained liver tissue sections were scanned by a Leica SCN 400 slide scanner (Leica Microsystems Ltd., Mannheim, Germany). The antibodies used for this study are listed in Table S1.

Immunofluorescent staining

Immunofluorescent co-staining was performed using 8-μm cryosections to observe the co-expression patterns of KRT19 and OV6, KRT7 and OV6, α-SMA and OV6, collagen type I (Col-I) and OV6, KRT19 and Gli1, KRT7 and Epcam, and KRT19 and α-SMA. For cellular immunofluorescent staining, 48-well plates with cells growing on coverslips were employed for detection of KRT19 and Gli1 expression. Staining of nuclei was performed with DAPI (1:1000, Beyotime Biotechnology, Shanghai, China). Cells were observed and photographed by a DP80 fluorescence inverted microscope (Olympus, Beijing, China) or laser scanning confocal microscope (Olympus, Beijing, China). The antibodies used for this study are listed in Table S1.

Western blot and densitometric analysis

Liver tissue (20-50 μg) was used for Western blot analysis, homogenized with RIPA buffer (Beyotime Biotechnology, P10013B, Shanghai, China) supplemented with protease and phosphatase inhibitor cocktail (Beyotime Biotechnology, P1045). For cellular experiments, 6-well plates were used for culture, and RIPA buffer was added to lyse cells. An Odyssey 2.1 software of Odyssey infrared scanner (LI-COR Biosciences, Lincoln, USA) was employed for scanning, and Image-J software was used for densitometric analysis. All antibodies used for this study are listed in Table S1.

Quantitative reverse transcript polymerase chain reaction (qRT-PCR)

Total RNA from frozen liver tissues or cells was isolated by MagExtractor Total RNA Purification Kit (TOYOBO, NPK-201, Shanghai, China). ReverTra Ace qPCR RT Master Mix with gDNA Remover (TOYOBO, FSQ-301) was used for reverse transcription. SYBR Green Realtime PCR Master Mix (TOYOBO, QPK-201) was employed for qRT-PCR. The primer sequences used are listed in Table S2.

RNA sequencing and bioinformatic analysis

Total RNA was extracted and then used to constructed cDNA libraries. The libraries were sequenced on an Illumina Novasep 6000 platform and 150 bp paired-end reads were generated. FPKM of each gene was calculated using Cufflinks, and the read counts of each gene were obtained by HTSeq-count. Differential expression analysis was performed using the DESeq2 R (v3.2.0). p value < 0.05 and fold-change > 2 or fold-change < 0.5 was set as the threshold for significantly differential expression. Hierarchical cluster analysis of differentially expressed genes (DEGs) was performed to demonstrate expression pattern of genes in different groups and samples. GO enrichment and KEGG pathway enrichment analysis of DEGs were performed respectively using R based on the hypergeometric distribution. The transcriptome sequencing and analysis were conducted by OE Biotech Co., Ltd. (Shanghai, China).

Gene set enrichment analysis (GSEA)

GSEA was implemented on the Java GSEA platform. For each GO enrichment and KEGG pathway, genes involved were defined as a gene set, and a ranked list and a 'gene set' permutation were then generated. NES values were normalized enrichment scores. A p value < 0.05 was set as the threshold for the enriched and trusted gene set.

Data availability

All relevant data are available from the corresponding authors upon reasonable request. All the data supporting the findings of this study are available within this article, supplementary material files. RNA-Seq data needs to upload to GeneBank for public access when the manuscript is published.

Reference

1. Desmet VJ, Gerber M, Hoofnagle JH, Manns M, Scheuer PJ. Classification of chronic

hepatitis: diagnosis, grading and staging. Hepatology. 1994; 19: 1513-20.
2. Lauth M, Bergstrom A, Shimokawa T, Toftgard R. Inhibition of GLI-mediated transcription and tumor cell growth by small-molecule antagonists. Proc Natl Acad Sci U S A. 2007; 104: 8455-60.
3. Ikenaga N, Liu SB, Sverdlov DY, Yoshida S, Nasser I, Ke Q, et al. A new Mdr2(-/-) mouse model of sclerosing cholangitis with rapid fibrosis progression, early-onset portal hypertension, and liver cancer. Am J Pathol. 2015; 185: 325-34.
4. Tsuchida T, Lee YA, Fujiwara N, Ybanez M, Allen B, Martins S, et al. A simple diet- and chemical-induced murine NASH model with rapid progression of steatohepatitis, fibrosis and liver cancer. J Hepatol. 2018; 69: 385-95.
5. Jamall IS, Finelli VN, Que Hee SS. A simple method to determine nanogram levels of 4-hydroxyproline in biological tissues. Anal Biochem. 1981; 112: 70-5.

Supplementary Figures

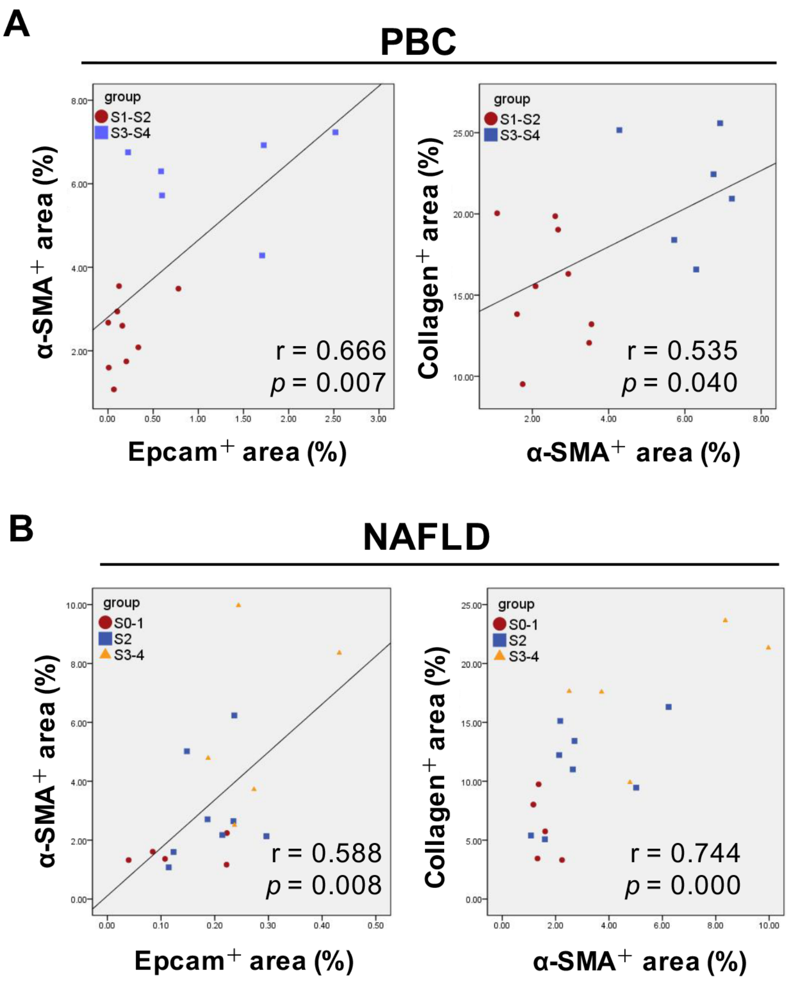

Figure S1. The correlation among α-SMA $^+$ area (%) and Epcam$^+$ area (%), collagen$^+$ area (%) and α-SMA $^+$ area (%) in patients with PBC or NAFLD. (A) patients with PBC. (B) patients with NAFLD. *, p < 0.05; **, p < 0.01. p values determined by Spearman's correlation analysis.

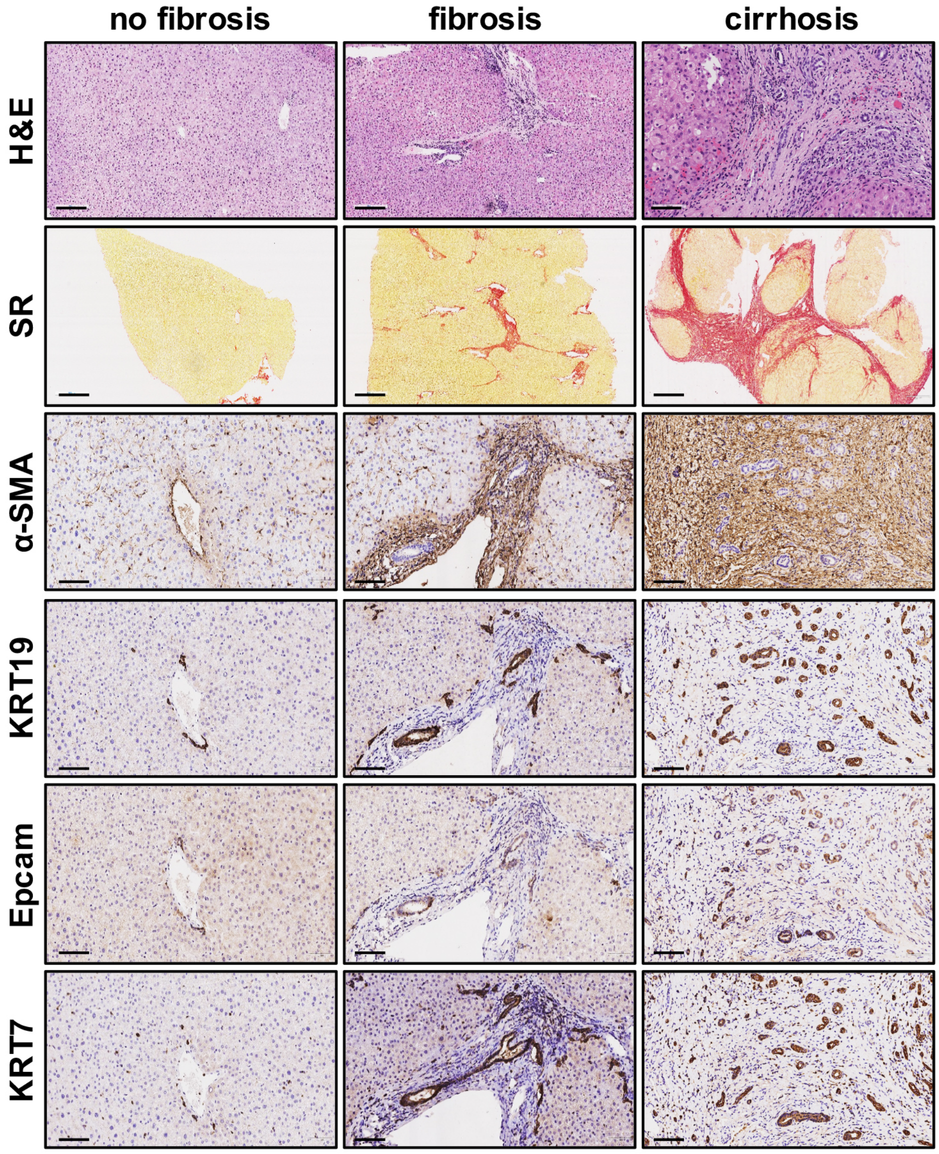

Figure S2. Representative H&E (scale bar = 100μm), SR (scale bar = 500μm), α-SMA (200×), KRT19 (scale bar = 100μm), Epcam (scale bar = 100μm) and KRT7 (scale bar = 100μm) staining of the hemangioma/paracancerous tissues of patients with hepatic hemangioma and HCC. (*n* = 3 per group).

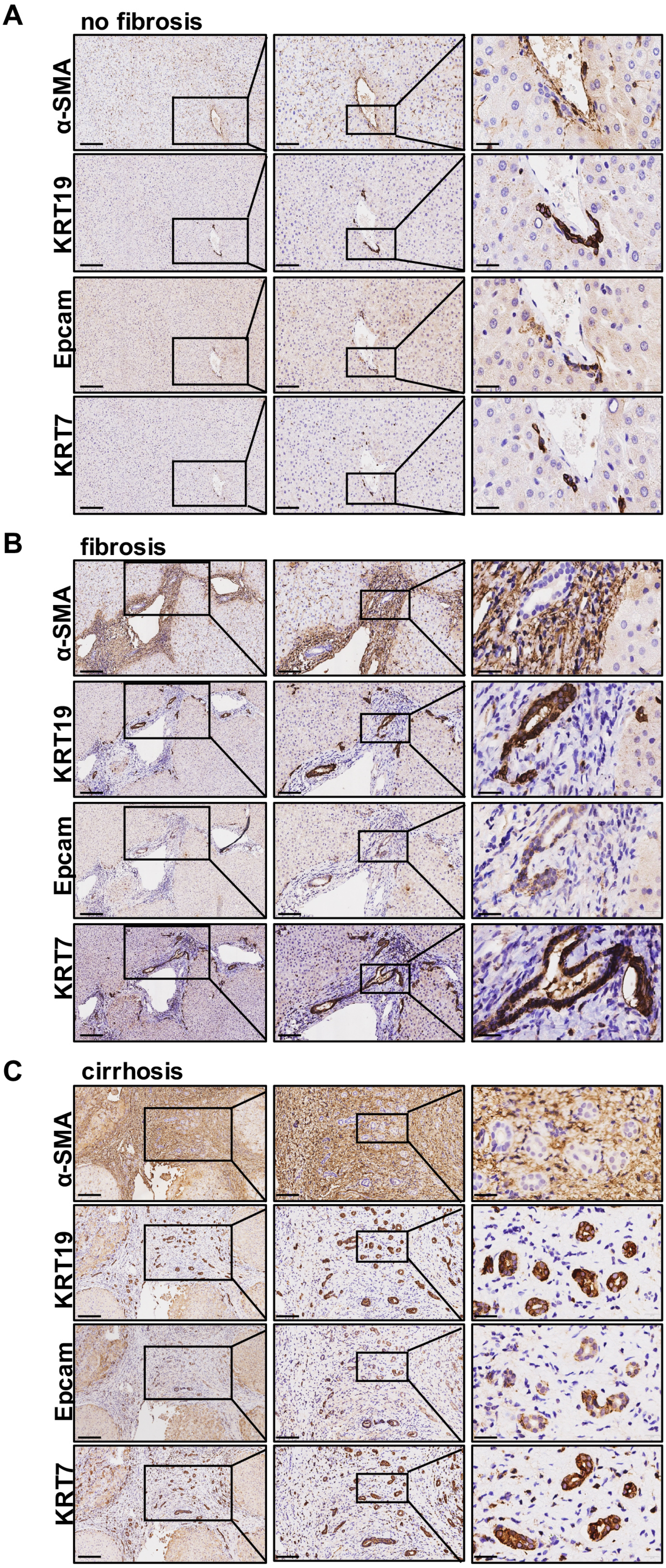
A
no fibrosis
α-SMA
KRT19
Epcam
KRT7
B
fibrosis
α-SMA
KRT19
Epcam
KRT7
C
cirrhosis
α-SMA
KRT19
Epcam
KRT7

Figure S3. The extended figures of immunohistochemical staining in hemangioma/paracancerous tissues of patients with hepatic hemangioma and HCC. (A) Representative images of liver sections in the non-fibrosis group stained with α-SMA, KRT19, Epcam, and KRT7 (scale bar = 100 μm, 50 μm and 25 μm). (B) Representative images of liver sections in the fibrosis group stained with α-SMA, KRT19, Epcam, and KRT7 (scale bar = 100 μm, 50 μm and 25 μm). (C) Representative images of liver sections in the cirrhosis group stained with α-SMA, KRT19, Epcam, and KRT7 (scale bar = 100 μm, 50 μm and 25 μm).

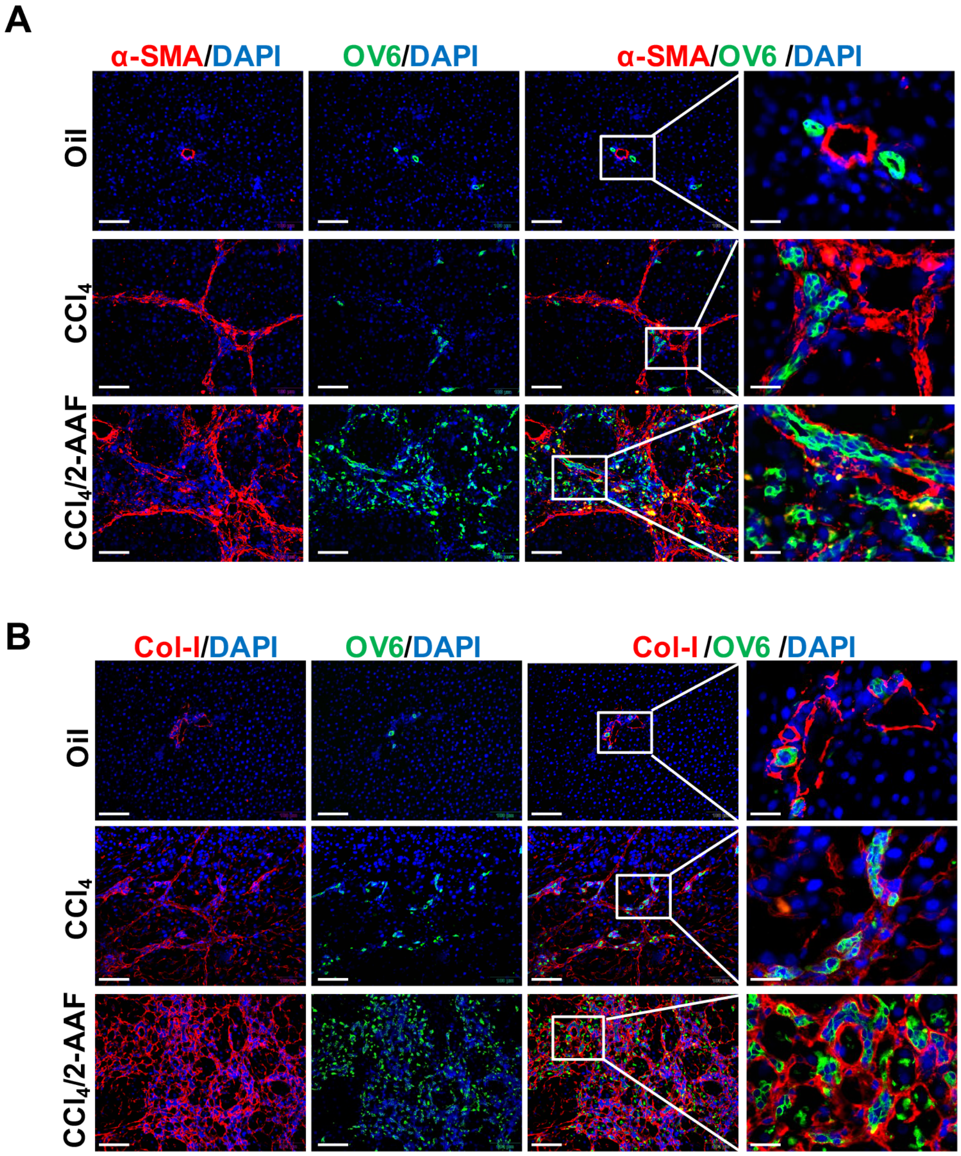

Figure S4. Confocal analysis of co-staining for α-SMA, Col I and OV6 in CCl_4/2-AAF-treated rats. (A) Confocal analysis of co-staining for α-SMA (red) and OV6 (green) (scale bar = 100 μm). (B) Confocal analysis of co-staining for Col-I (red) and OV6 (green) (scale bar = 100 μm). Nuclei counterstained with DAPI (blue). The right-most column in A and B is the higher magnification of the white box area (scale bar = 25 μm). Oil: the control group; CCl_4: the CCl_4-treated group; CCl_4/2-AAF: the CCl_4 combined with 2-AAF-treated group.

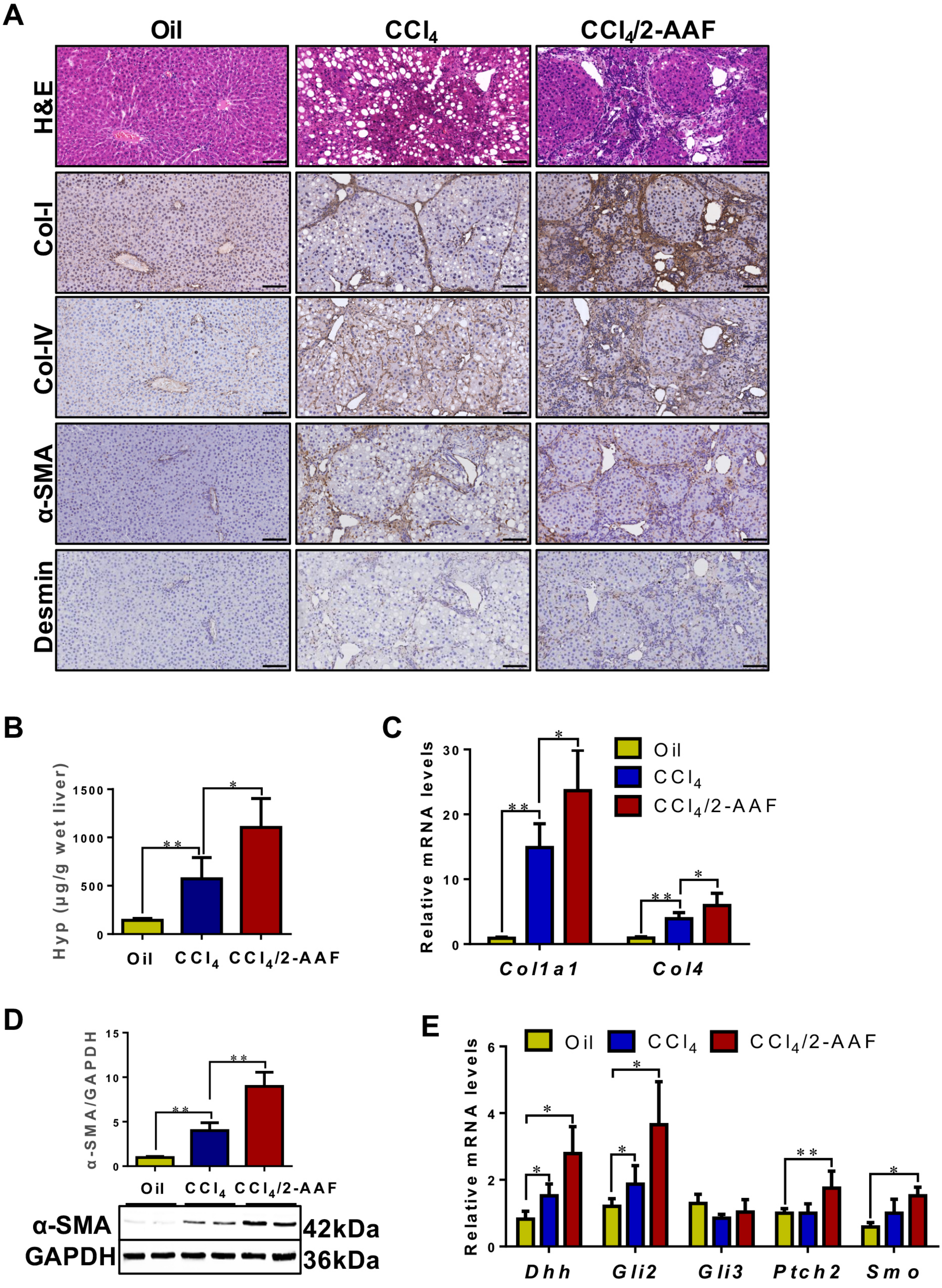

Figure S5. 2-AAF aggravates liver fibrosis during the progression of liver fibrosis. (A) Representative H&E, Col-I, Col-IV, α-SMA, and Desmin staining (scale bar = 100 μm). (B) Hepatic collagen content as determined biochemically via hydroxyproline. (D) Gene expression of *Col1a1* and *Col4* was determined by qRT-

PCR. mRNA values were normalized against *Gapdh* levels and are shown relative to expression level in the control group. (D) Immunoblotting for α-SMA. GAPDH was used as loading control. (E) Gene expressions of *Dhh, Gli2, Gli3, Ptch2* and *Smo* were determined by qRT-PCR. *, $p < 0.05$; **, $p < 0.01$. Oil: the control group; CCl_4: the CCl_4-treated group; CCl_4/2-AAF: the CCl_4 combined with 2-AAF-treated group.

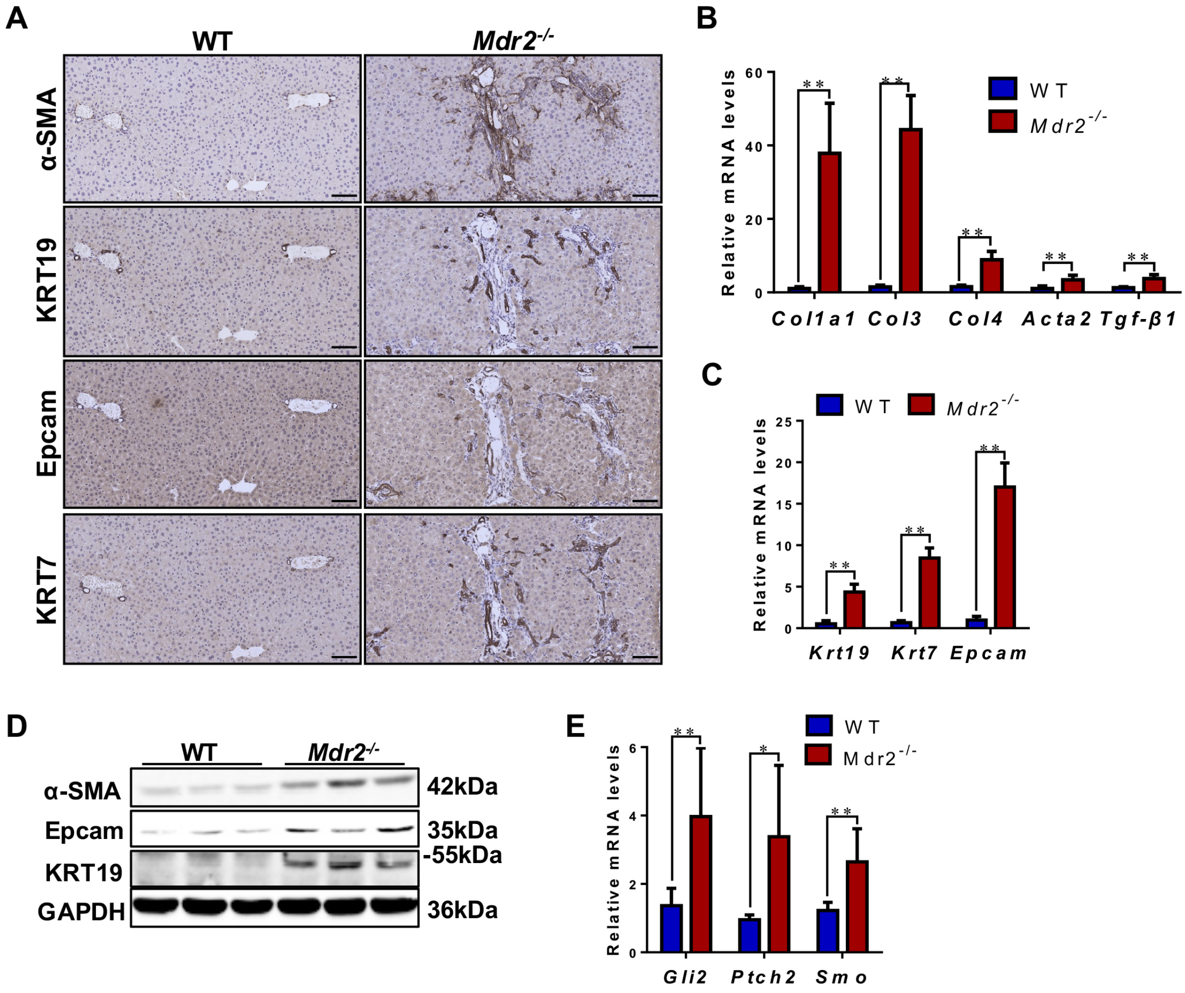

Figure S6. DR and Hedgehog signaling in *Mdr2*$^{-/-}$ mice. (A) Representative images of liver sections stained with α-SMA, KRT19, Epcam, and KRT7 (scale bar = 100 μm). (B) Gene expression of *Col1a1*, *Col3*, *Col4*, *Acta2*, and *Tgf-β1* was determined by qRT-PCR. (C) Gene expression of *Krt19, Krt7* and *Epcam* was determined by qRT-PCR. (D) Immunoblotting for α-SMA, Epcam and KRT19. GAPDH was used as loading control. (E) Gene expression of *Gli2*, *Ptch2* and *Smo* was determined by qRT-PCR. All mRNA values were normalized against *Gapdh* levels and are shown relative to expression level in the control group. *, $p < 0.05$; **, $p < 0.01$. WT: the wild-type mice; *Mdr2*$^{-/-}$: the *Mdr2*$^{-/-}$ mice.

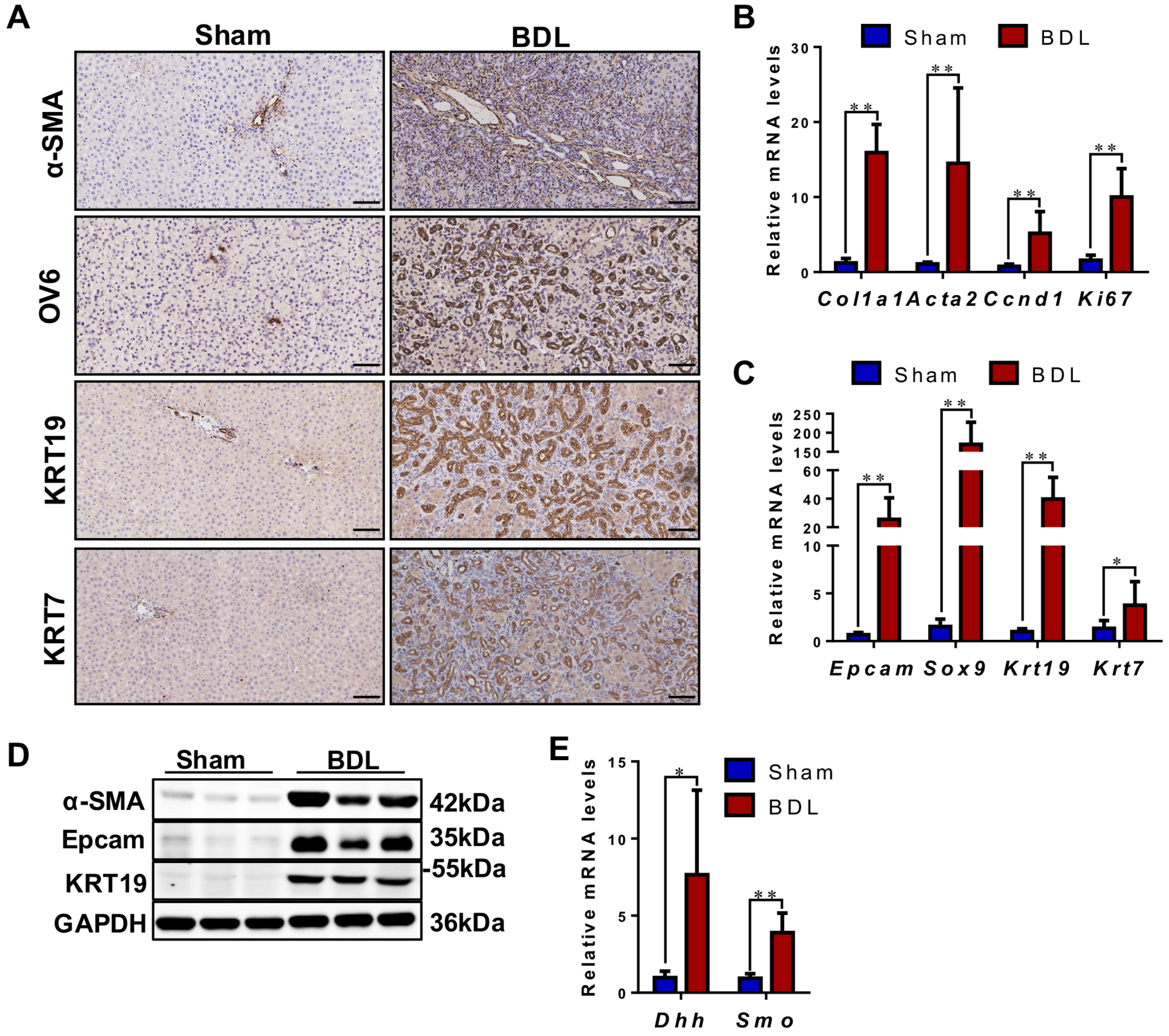

Figure S7. DR and Hedgehog signaling pathway in rats with BDL. (A) Representative images of liver sections stained with α-SMA, OV6, KRT19, and KRT7 (scale bar = 100 μm). (B) Gene expression of *Col1a1*, *Acta2*, *Ccnd1*, and *Ki67* was determined by qRT-PCR. (C) Gene expression of *Epcam*, *Sox9, Krt19* and *Krt7* was determined by qRT-PCR. (D) Immunoblotting for α-SMA, Epcam, and KRT19. GAPDH was used as loading control. (E) Gene expression of *Dhh* and *Smo* was determined by qRT-PCR. All mRNA values were normalized against *Gapdh* levels and are shown relative to expression level in the control group. *, $p < 0.05$; **, $p < 0.01$. Sham: the sham-operated group; BDL: the bile duct ligation group.

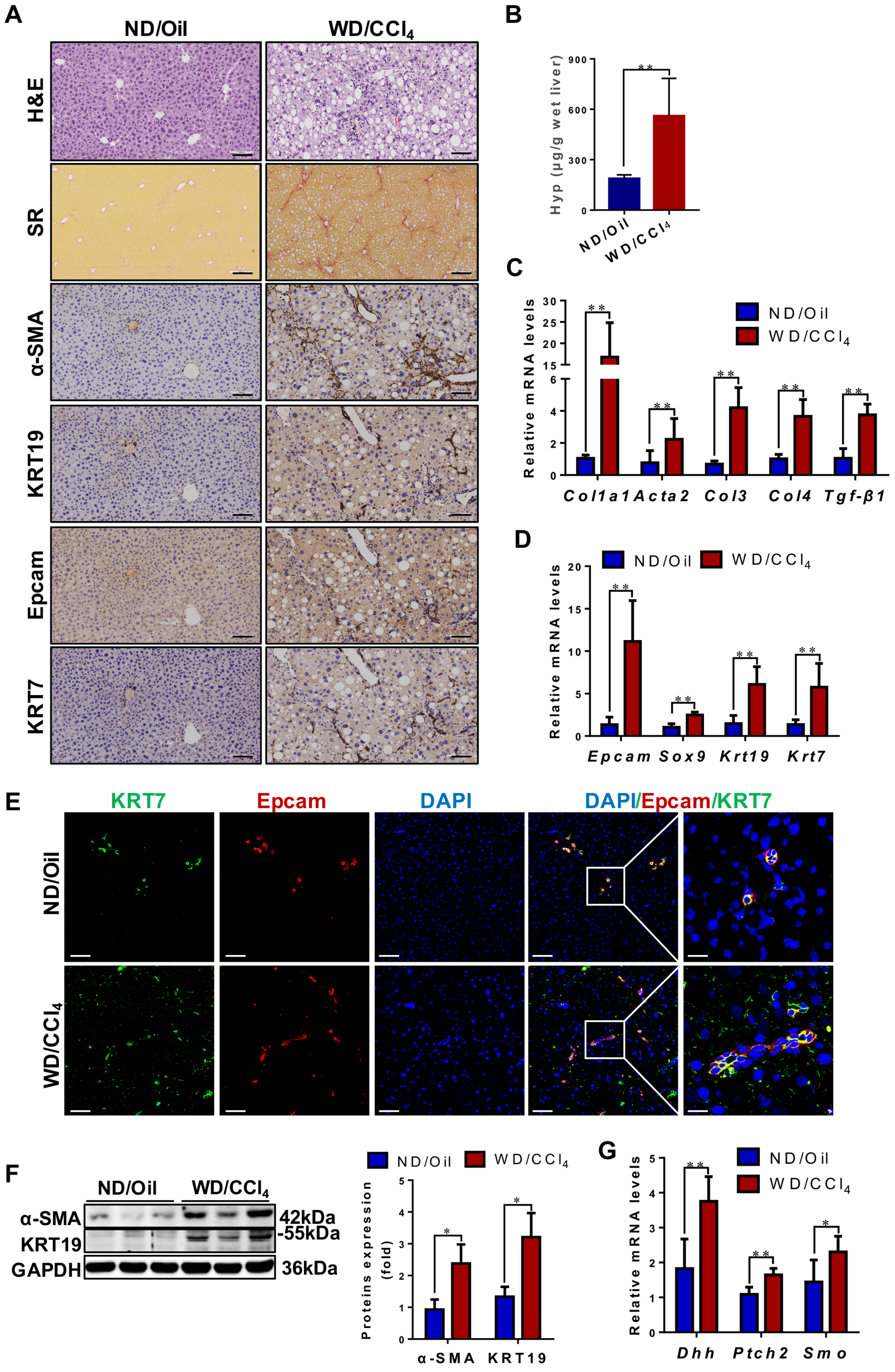
A
ND/Oil
WD/CCl4
H&E
SR
α-SMA
KRT19
Epcam
KRT7
B
Hyp (μg/g wet liver)
ND/Oil
WD/CCl4
C
Relative mRNA levels
ND/Oil
WD/CCl4
Col1a1
Acta2
Col3
Col4
Tgf-β1
D
Relative mRNA levels
Epcam
Sox9
Krt19
Krt7
E
KRT7
Epcam
DAPI
DAPI/Epcam/KRT7
F
α-SMA
KRT19
GAPDH
42kDa
-55kDa
36kDa
Proteins expression (fold)
G
Dhh
Ptch2
Smo

Figure S8. DR and Hedgehog signaling pathway in WD-fed/CCl_4-treated mice. (A) Representative images of liver sections stained with H&E (scale bar = 100 μm), SR (scale bar = 50 μm), α-SMA (scale bar = 100 μm), KRT19 (scale bar = 100μm), Epcam (scale bar = 100 μm), and KRT7 (scale bar = 100 μm). (B) Hepatic collagen content as determined biochemically via Hyp. (C) Gene expression of *Col1a1*, *Acta2*, *Col3*, *Col4*, and *Tgf-β1* was determined by qRT-PCR. (D) Gene expression of *Epcam*, *Sox9, Krt19* and *Krt7* was determined by qRT-PCR. (E) Confocal analysis of co-staining for KRT7 (green) and Epcam (red) (scale bar = 100 μm). Nuclei counterstained with DAPI (blue). The right-most column is the higher magnification of the white box area (scale bar = 25 μm). (F) Immunoblotting and quantification of α-SMA and KRT19. GAPDH was used as loading control. (G) Gene expressions of *Dhh*, *Ptch2* and *Smo* were determined by qRT-PCR. All mRNA values were normalized against *Gapdh* levels and are shown relative to expression level in the control group. *, $p < 0.05$; **, $p < 0.01$. ND/oil: the control group; WD/CCl_4: the western diet-fed and CCl_4-treated group.

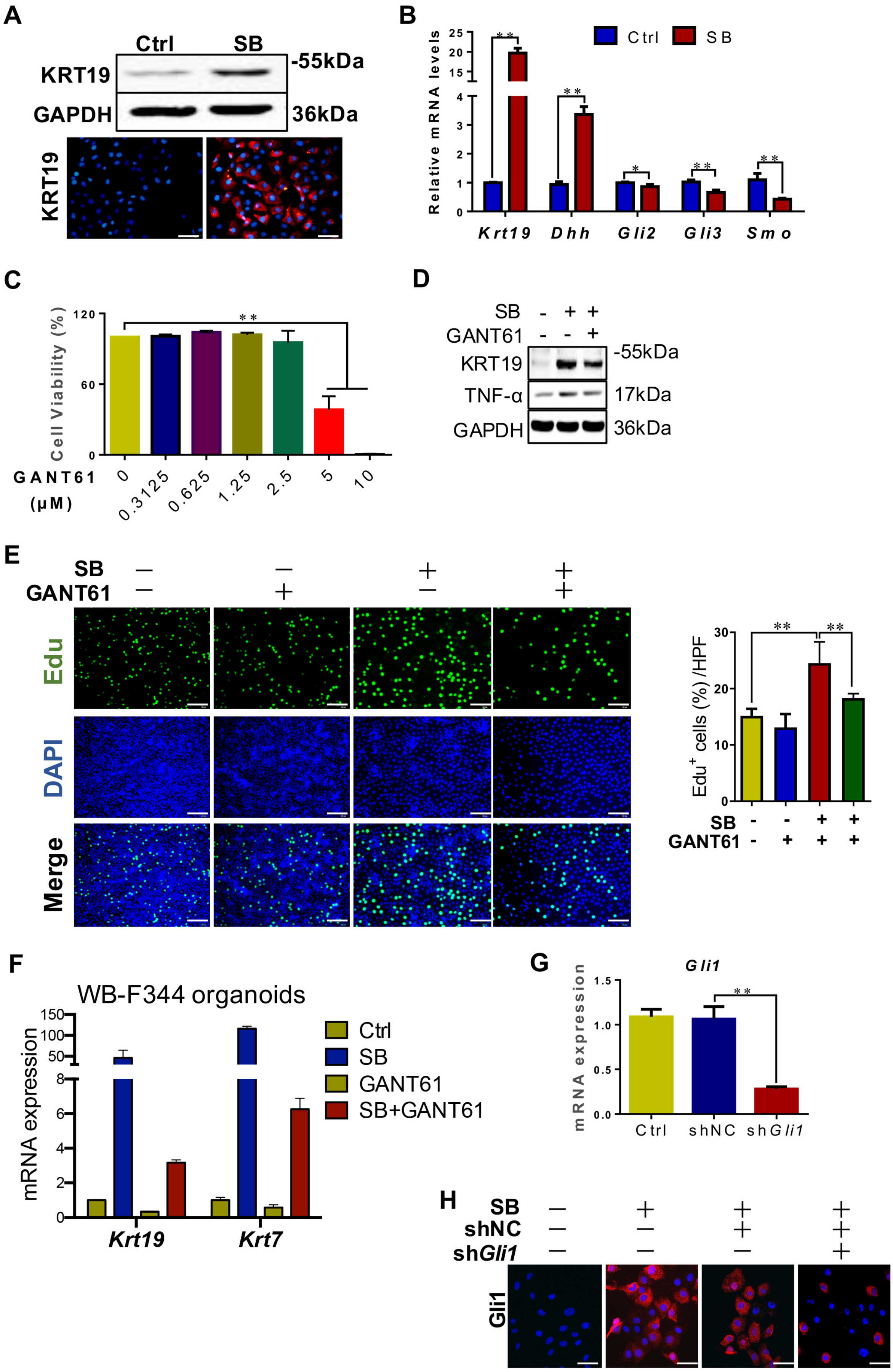

A
Ctrl
SB
KRT19
-55kDa
GAPDH
36kDa
KRT19
B
Ctrl
SB
Relative mRNA levels
Krt19
Dhh
Gli2
Gli3
Smo
C
Cell Viability (%)
GANT61
(μM)
0
0.3125
0.625
1.25
2.5
5
10
D
SB
GANT61
KRT19
-55kDa
TNF-α
17kDa
GAPDH
36kDa
E
SB
GANT61
Edu
DAPI
Merge
Edu+ cells (%) /HPF
F
WB-F344 organoids
mRNA expression
Ctrl
SB
GANT61
SB+GANT61
Krt19
Krt7
G
Gli1
mRNA expression
Ctrl
shNC
shGli1
H
SB
shNC
shGli1
Gli1

Figure S9. Expression of Gli1 signaling pathway during the differentiation of WB-F344 cells into cholangiocytes. (A) Western blot and immunofluorescence staining of KRT19 (scale bar = 100 μm). (B) Gene expression of *Krt19*, *Dhh*, *Gli2*, *Gli3*, and *Smo* was determined by qRT-PCR. (C) The effect of GANT61 on the viability of WB-F344 cells. (D) Immunoblotting for KRT19 and TNF-α. GAPDH was used as loading control. (E) Representative images of WB-F344 cells stained with Edu (scale bar = 100 μm) with or without GANT61 and Edu^+ cells per HPF. (F) Gene expression of *Krt19* and *Krt7* of WB-F344 organoids. (G) *Gli1* mRNA expression was determined by qRT-PCR. All mRNA values were normalized against *Gapdh* levels and are shown relative to expression level in the control group. (H) Representative images of WB-F344 cells stained with Gli1 after *Gli1* knockdown (scale bar = 33.3 μm). *, $p < 0.05$; **, $p < 0.01$. Ctrl: the control group; SB: the sodium butyrate-treated group; shNC: the negative lentivirus group; sh*Gli1*: the *Gli1* knockdown group.

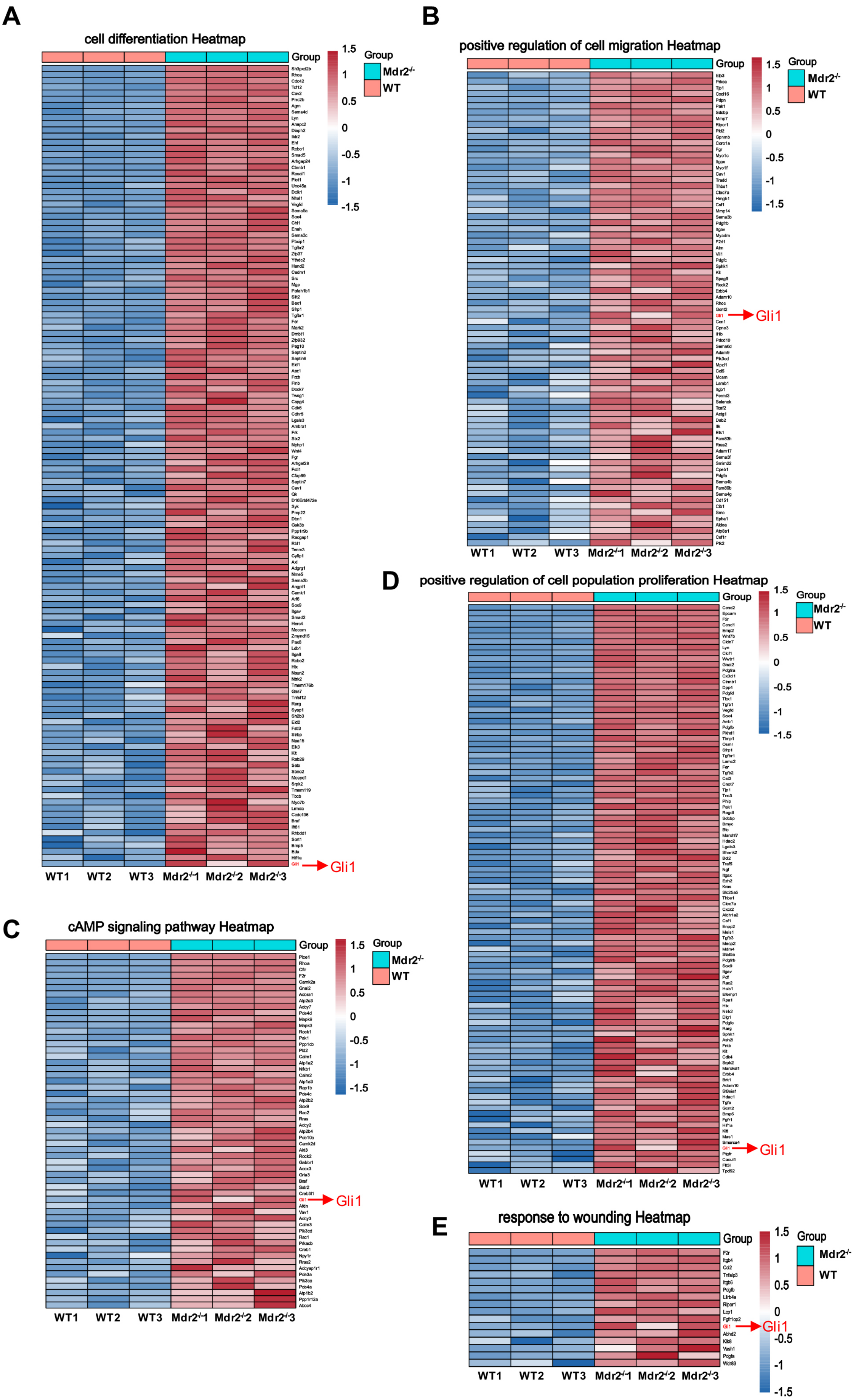
A
cell differentiation Heatmap
Group
Mdr2-/-
WT
1.5
1
0.5
0
-0.5
-1
-1.5
Gli1
WT1 WT2 WT3 Mdr2-/-1 Mdr2-/-2 Mdr2-/-3
B
positive regulation of cell migration Heatmap
Group
Mdr2-/-
WT
Gli1
WT1 WT2 WT3 Mdr2-/-1 Mdr2-/-2 Mdr2-/-3
C
cAMP signaling pathway Heatmap
Group
Mdr2-/-
WT
Gli1
WT1 WT2 WT3 Mdr2-/-1 Mdr2-/-2 Mdr2-/-3
D
positive regulation of cell population proliferation Heatmap
Group
Mdr2-/-
WT
Gli1
WT1 WT2 WT3 Mdr2-/-1 Mdr2-/-2 Mdr2-/-3
E
response to wounding Heatmap
Group
Mdr2-/-
WT
Gli1
WT1 WT2 WT3 Mdr2-/-1 Mdr2-/-2 Mdr2-/-3

Figure S10. Heat map of the significantly enriched items in livers of *Mdr2*$^{-/-}$ mice. (A) Heat map of cell differentiation. (B) Heat map of positive regulation of cell migration. (C) Heat map of cAMP signaling pathway. (D) Heat map of positive regulation of cell population differentiation. (E) Heat map of response to wounding. Heat map showed that Gli1 expression increased in all of these significantly enriched items (red arrow).

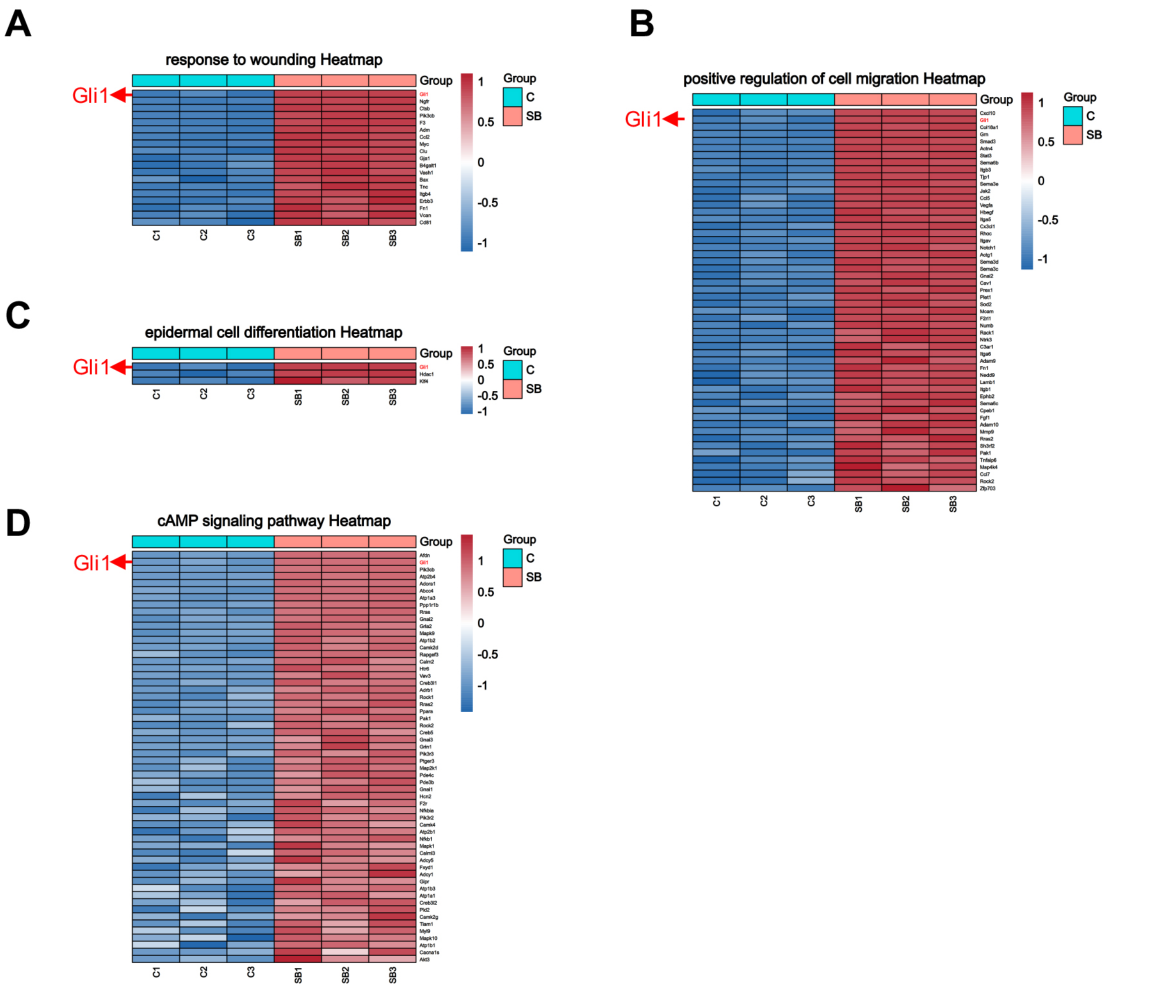

Figure S11. Heat map of the significantly enriched items in WB-F344 cells, treated with or without SB. (A) Heat map of response to wounding. (B) Heat map of positive regulation of cell migration. (C) Heat map of epidermal cell differentiation. (D) Heat map of cAMP signaling pathway. Heat map showed that Gli1 expression increased in all of these significantly enriched items (red arrow).

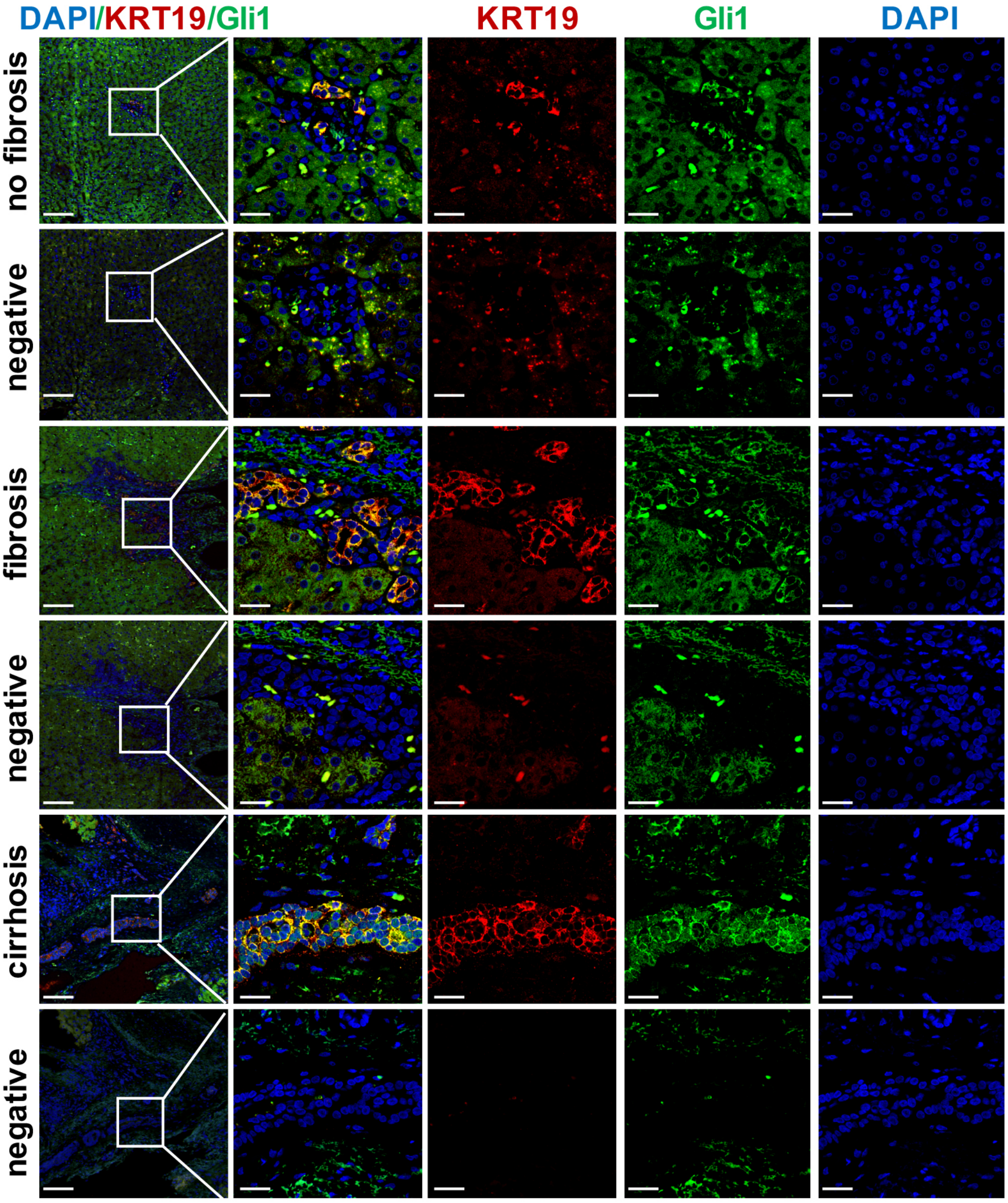

Figure S12. The extended figures of Figure 4E. Confocal analysis of co-staining for KRT19 (red) and Gli1 (green) of paraffin sections in hemangioma/paracancerous tissues of patients with hepatic hemangioma and HCC (scale bar = 100 μm and 25 μm). Nuclei counterstained with DAPI (blue). Negative pictures showed the serial sections with negative staining.

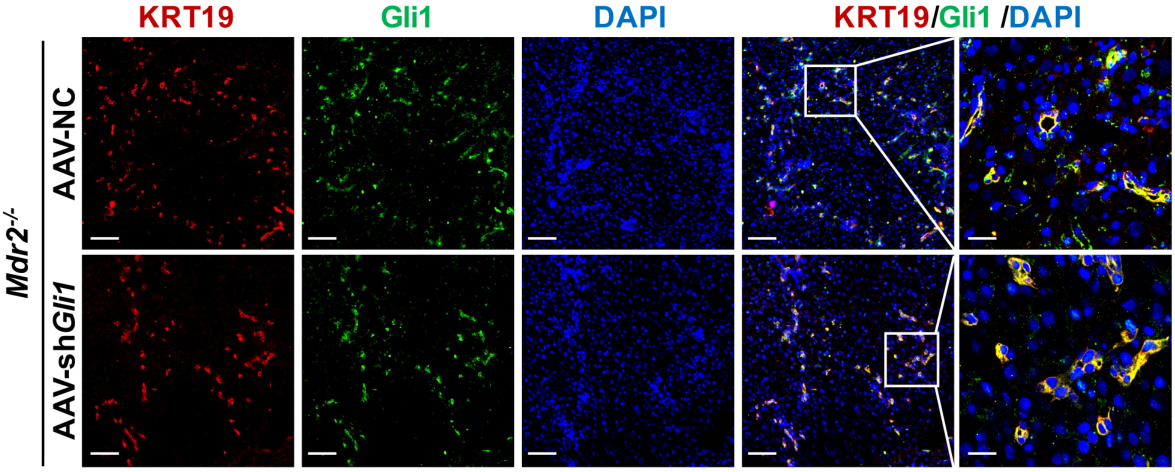

Figure S13. Confocal analysis of co-staining for KRT19 (red) and Gli1 (green) of frozen sections of *Mdr2*$^{-/-}$ mice after injected with AAV-NC or AAV-sh*Gli1* (scale bar = 100 μm)**.** Higher magnification of the white box area (scale bar = 25 μm) is shown in the right-most column. Nuclei counterstained with DAPI (blue).

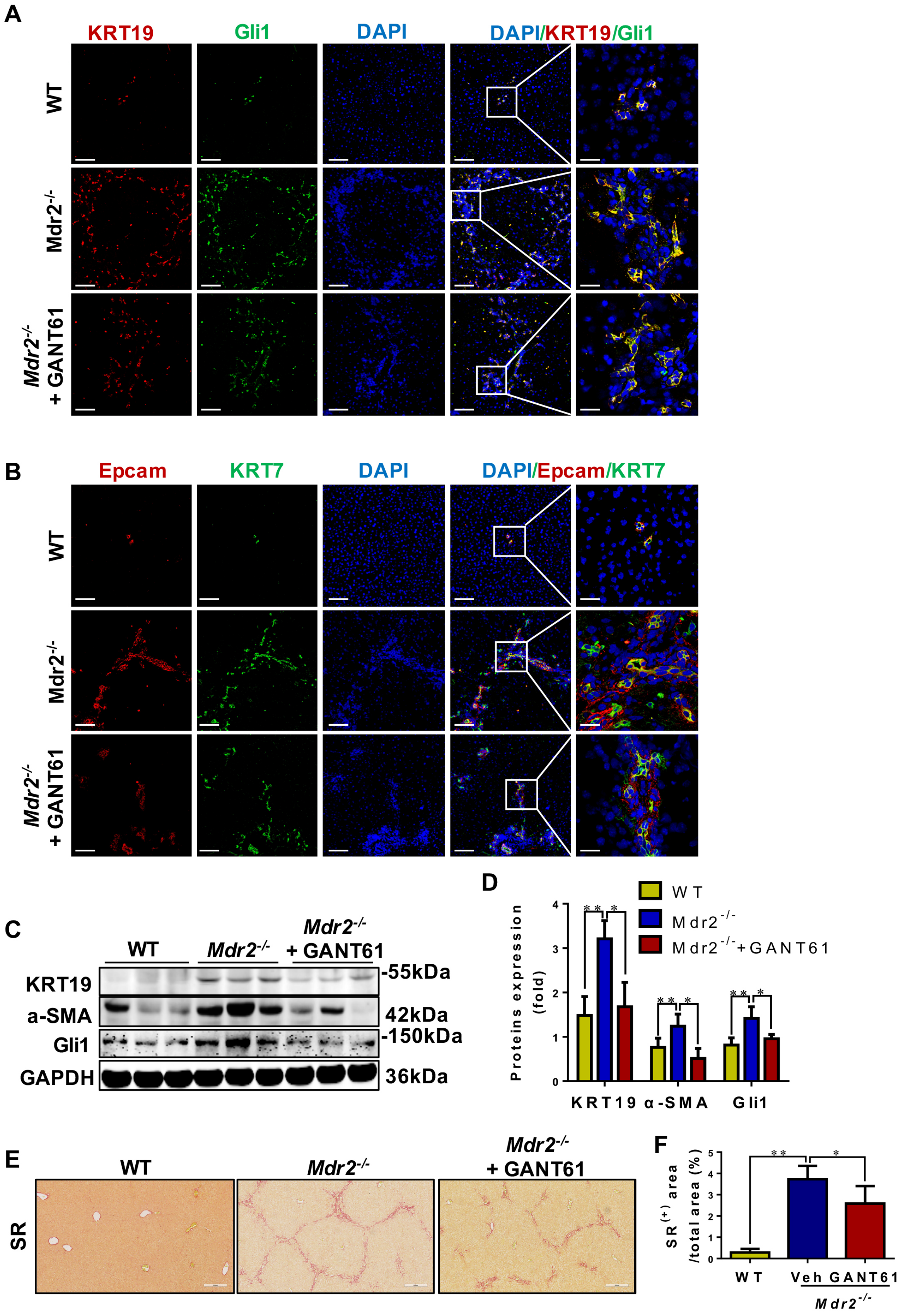

Figure S14. The effect of GANT61 on DR and the degree of hepatic fibrosis in *Mdr2*$^{-/-}$ mice. (A) Confocal analysis of co-staining for KRT19 (red) and Gli1 (green) (scale bar = 100 μm). (B) Confocal analysis of co-staining for Epcam (red) and KRT7

(green) (scale bar = 100 μm). Nuclei counterstained with DAPI (blue). The right-most column in A and B is the higher magnification of the white box area (scale bar = 25 μm). (C) and (D) Immunoblotting for KRT19, α-SMA and Gli1. The quantification of KRT19, α-SMA and Gli1 were measured employing histogram normalized to GAPDH protein based on the results of Western blot (n = 3 per group). (E) Representative images of liver sections stained with SR (scale bar = 200 μm). (F) Collagen morphometry (%) of SR^+ area (n = 6 per group). *, $p < 0.05$; **, $p < 0.01$. WT, the wild-type group; *Mdr2*$^{-/-}$, the *Mdr2*$^{-/-}$ group; *Mdr2*$^{-/-}$ + GANT61, the GANT61-treated group.

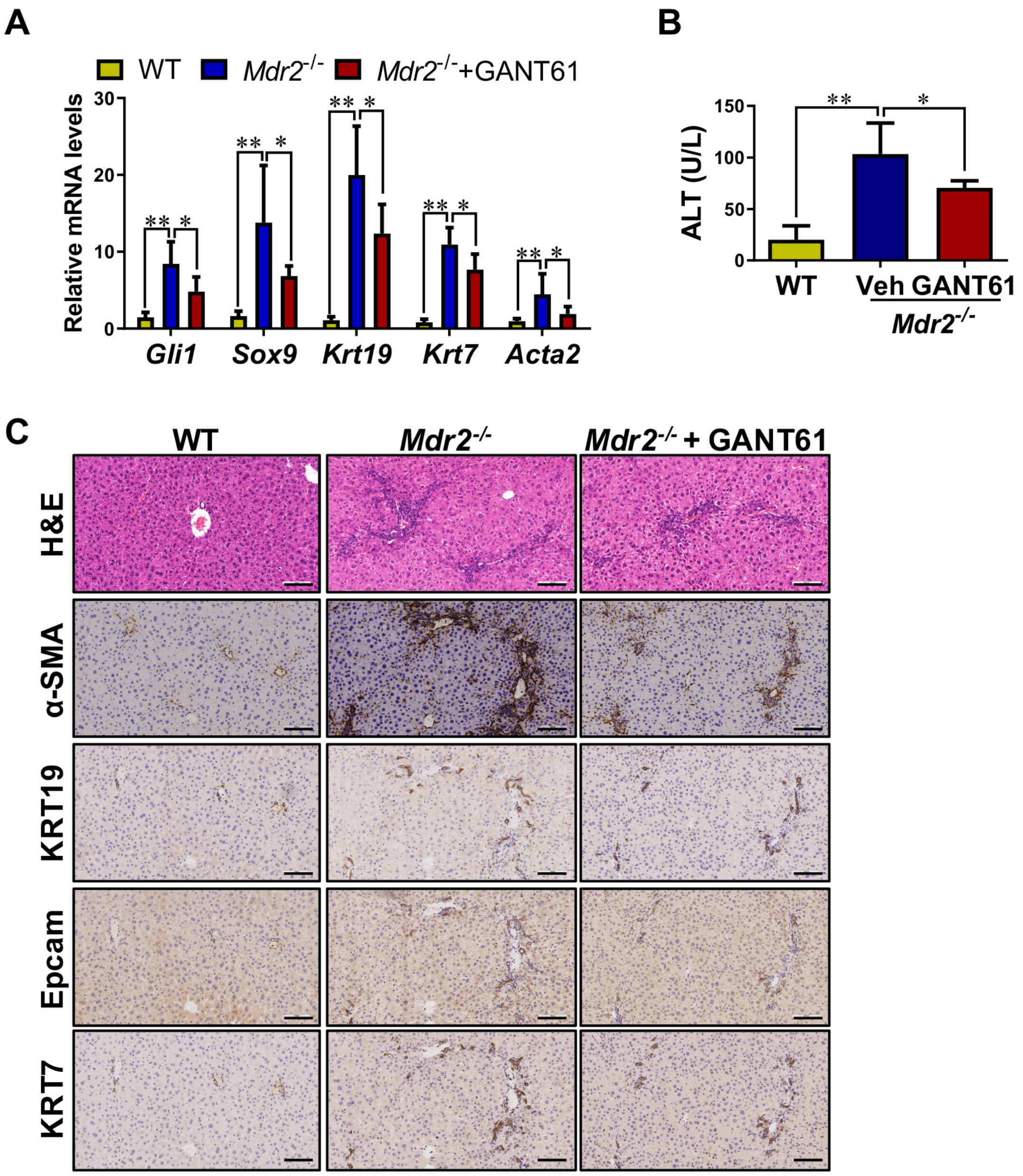

Figure S15. The effect of GANT61 on DR and the degree of hepatic fibrosis in *Mdr2*$^{-/-}$ mice. (A) Gene expression of *Gli1*, *Sox9*, *Krt19*, *Krt7*, and *Acta2* was determined by qRT-PCR ($n = 6$ per group). (B) Serum ALT activity. (C) Representative images of liver sections stained with H&E, α-SMA, KRT19, Epcam and KRT7 (scale bar = 100 μm). *, $p < 0.05$; **, $p < 0.01$. WT, the wild-type group; *Mdr2*$^{-/-}$, the *Mdr2*$^{-/-}$ group; *Mdr2*$^{-/-}$ + GANT61, the GANT61-treated group.

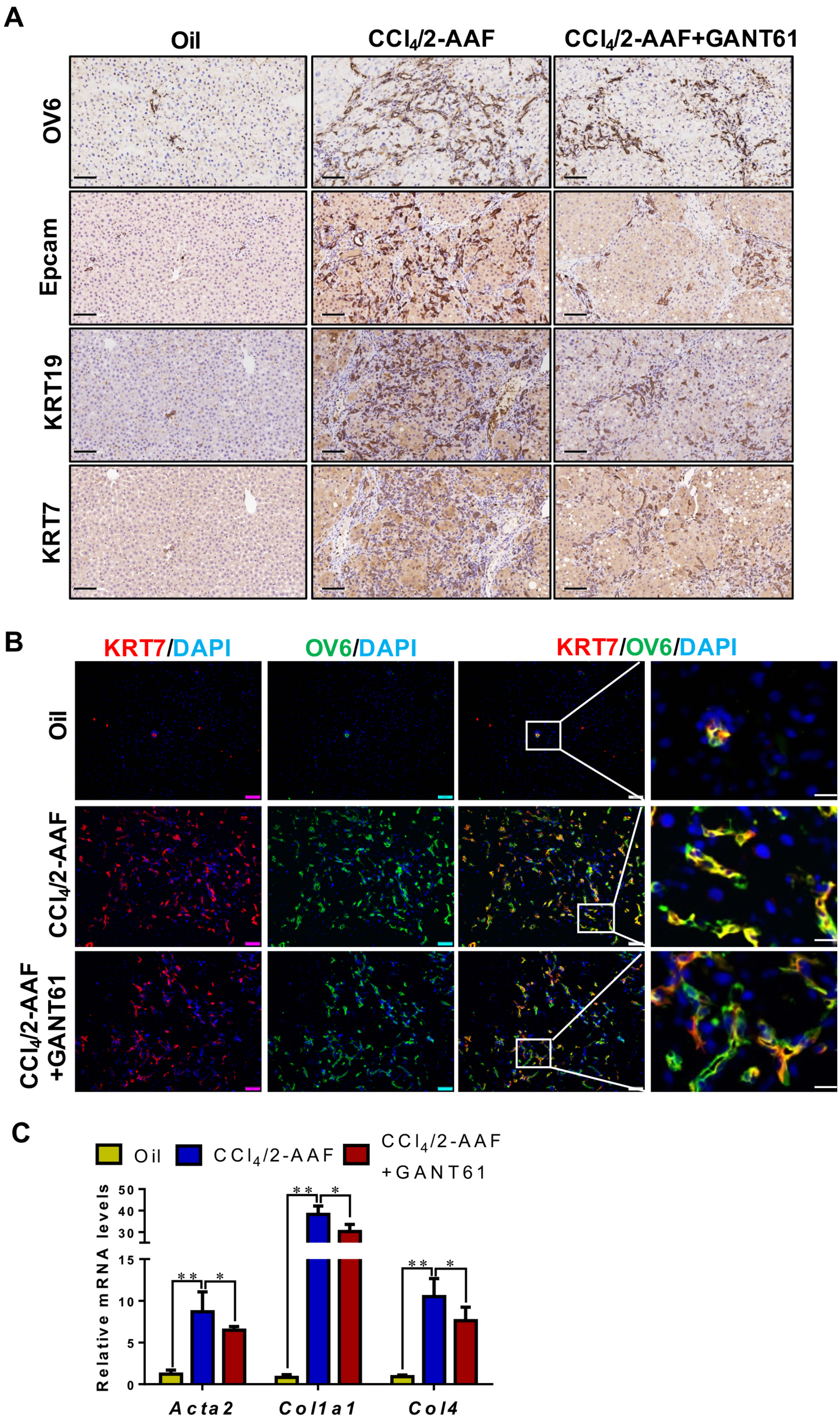
A
Oil
CCl4/2-AAF
CCl4/2-AAF+GANT61
OV6
Epcam
KRT19
KRT7
B
KRT7/DAPI
OV6/DAPI
KRT7/OV6/DAPI
Oil
CCl4/2-AAF
CCl4/2-AAF
+GANT61
C
Oil
CCl4/2-AAF
CCl4/2-AAF
+GANT61
Relative mRNA levels
0
5
10
15
30
40
50
**
*
Acta2
Col1a1
Col4

Figure S16. The effects of GANT61 on DR and liver fibrosis in CCl_4/2-AAF-treated rats. (A) Representative images of liver sections stained with OV6, Epcam, KRT19 and KRT7 (scale bar = 100 μm). (B) Confocal analysis of co-staining for KRT7 (red), OV6 (green) and DAPI (blue) of frozen sections (scale bar = 100 μm). The right-most column is the higher magnification of the white box area (scale bar = 25 μm). (C) Gene expression of *Acta2, Col1a1*, and *Col4* was determined by qRT-PCR. mRNA values were normalized against *Gapdh* levels and were shown relative to expression level in the control group. *, $p < 0.05$; **, $p < 0.01$. Oil: the control group; CCl_4/2-AAF: the CCl_4 combined with 2-AAF-treated group; CCl_4/2-AAF + GANT61: the GANT61-treated group.

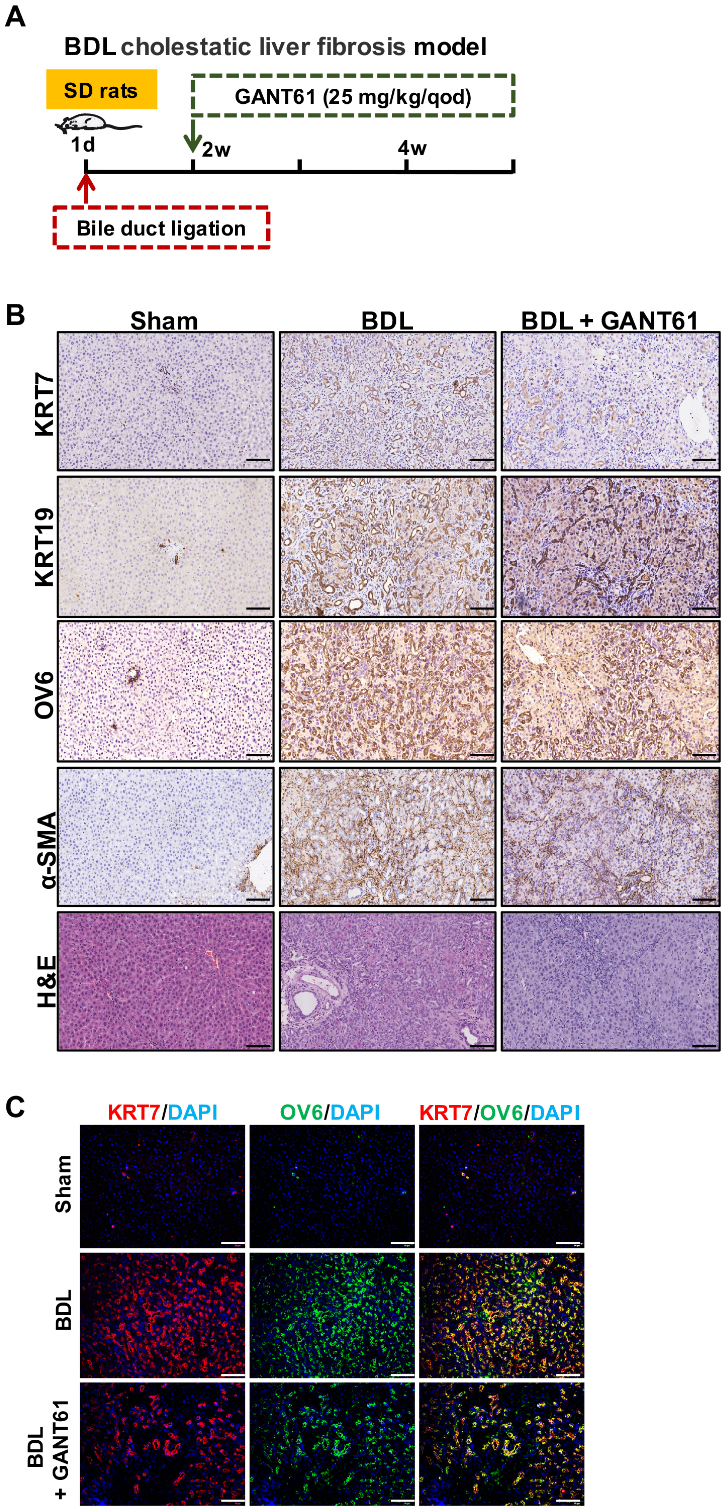

Figure S17. The effect of GANT61 on the degree of liver fibrosis in rats with BDL. (A) Schematic diagram of the experimental design of treatment with GANT61 in BDL rats. (B) Representative images of liver sections stained with KRT7, KRT19, OV6, α-SMA and H&E (scale bar = 100 μm). (C) Confocal analysis of co-staining for KRT7 (red), OV6 (green) and DAPI (blue) (scale bar = 100 μm). Sham: the sham-operated group; BDL: the bile duct ligation group; BDL + GANT61: the GANT61-treated group.

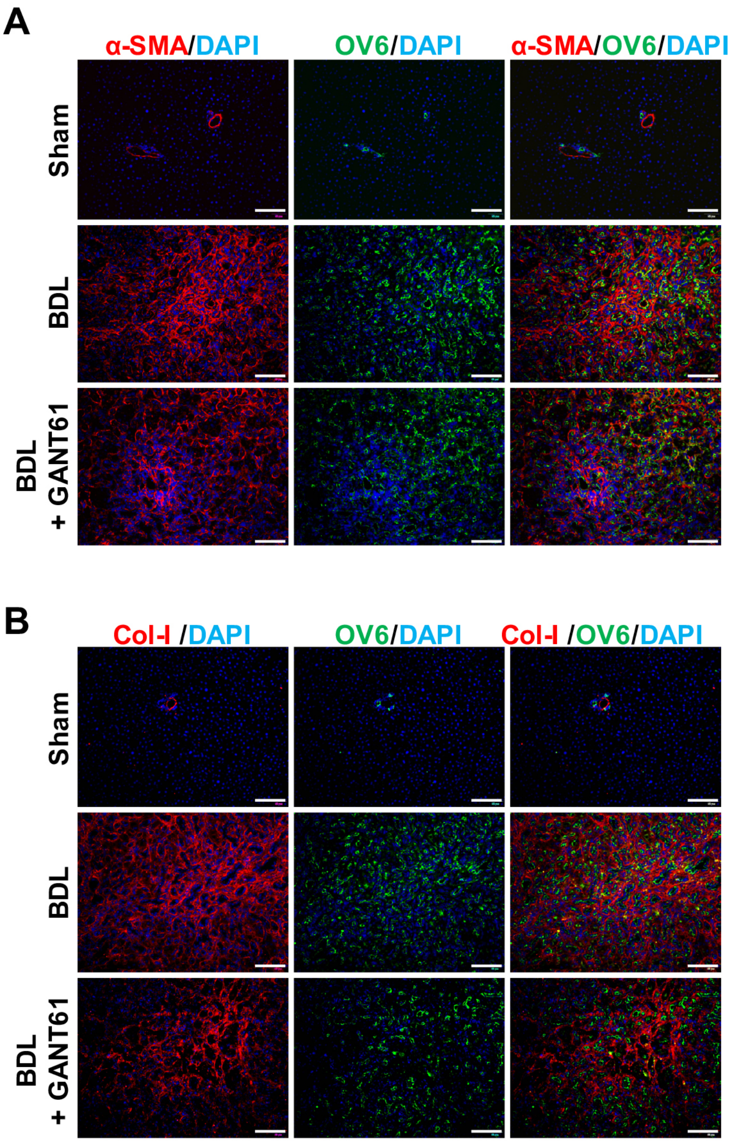

Figure S18. The effect of GANT61 on the degree of liver fibrosis in rats with BDL. (C) Confocal analysis of co-staining for α-SMA (red) and OV6 (green) (scale bar = 100 μm). (D) Confocal analysis of co-staining for Col-I (red) and OV6 (green) (scale bar = 100 μm). Nuclei counterstained with DAPI (blue). Sham: the sham-operated group; BDL: the bile duct ligation group; BDL + GANT61: the GANT61-treated group.

Supplementary Tables

Table S1. Antibodies used for immunostaining. IHC, Immunohistochemistry; IF, Immunofluorescence; WB, Western Blot.

Application	Specificity	Catalog#	Vendor	Dilutio
IHC	α-SMA	ab124964	Abcam	1:1000
IHC	Desmin	ab15200	Abcam	1:200
IHC	Col-IV	ab6586	Abcam	1:400
IHC	Col-I	ab34710	Abcam	1:200
IHC	KRT19	10712-1-AP	Proteintech	1:200
IHC	KRT7	15339-1-AP	Proteintech	1:200
IHC	Epcam	ab71916	Abcam	1:800
IHC	OV6	sc-101863	Santa Cruz	1:400
IF	α-SMA	ab124964	Abcam	1:1000
IF	α-SMA	ab7817	Abcam	1:200
IF	Col-I	ab34710	Abcam	1:200
IF	KRT19	10712-1-AP	Proteintech	1:200
IF	KRT7	15339-1-AP	Proteintech	1:200
IF	Epcam	ab71916	Abcam	1:800
IF	OV6	sc-101863	Santa Cruz	1:400
IF	Gli1	AF3455	R&D	1:50
IF	Gli1	A14965	Abclonal	1:200
IF	DAPI	C1002	Beyotime Biotechnology	1:1000
IF	Goat Anti-Rabbit IgG H&L (Cy3 ®)	ab6939	Abcam	1:3000
IF	Goat anti-mouse IgG H&L(FITC)	ab6785	Abcam	1:3000
IF	Donkey anti-Goat IgG H&L(Alexa Fluor ®488)	ab150129	Abcam	1:1000
WB	α-SMA	ab5694	Abcam	1:500
WB	KRT19	10712-1-AP	Proteintech	1:1000
WB	TNFα	BS1857	Bioworld	1:1000
WB	Epcam	ab71916	Abcam	1:1000
WB	Gli1	A14965	Abclonal	1:1000
WB	GAPDH	60004-1-AP	Proteintech	1:1000
WB	Anti-mouse IgG (H+L) (DyLight™ 800 4X PEG Conjugate)	5257	CST	1:1000
WB	Anti-rabbit IgG (H+L) (DyLight™ 680 Conjugate)	5366	CST	1:1000

Table S2. qRT-PCR Primers Used in this Study.

Gene	Primers Forward (5"to3")	Primers Reverse (5"to3")
Rat *Sox9*	GGAGCGACAACTTTACCAG	AGGGAAAACAGAGAACGAAA
Rat *Epcam*	GGCGTGGAACTCAGAACTTA	TCTACTGTGGGCTGTTTATG
Rat *Ki67*	GAGAACCCTGTCACTCCAGATCA	GGATTCTGCGGCTCTGTCTC
Rat *Ccnd1*	TACCGCACAACGCACTTTC	AAGGGCTTCAATCTGTTCCTG
Rat *Krt19*	CAGGTCGCTGTCCACACTA	TATCTCTGCCACAGTGCCTT
Rat *Krt7*	CGGAATGAGATTGCGGAGAT	CCTTGTTCCTCAGCCTCTGC
Rat *Col1a1*	TGTCTGGTTTGGAGAGAGCA	AGTGATAGGTGATGTTCTGG
Rat *Col4*	TTTCCAGGGTTACAAGGTGT	AGTCCAGGTTCTCCAGCATC
Rat *Acta2*	AGACCTTCAATGTCCCTGCCA	GTTGTGAGTCACGCCATCTCC
Rat *Gli1*	AGCATCACCGAAAATGTTG	TATCCCAGAGTGTCAGCAGA
Rat *Dhh*	GCCCCAATCTGTCAGGAATG	AAGTCCCACCCCACTCAAAG
Rat *Smo*	TCCAGCGAGACCCTATCCT	AACCACACTACTCCAGCCAT
Rat *Ptch2*	TAGTCTGCCGCTGCCAACTT	GCCCTGTGAGGTCTCTGTGA
Rat *Gli2*	GACCTGACCCCCTTGTTCT	CAGCGGACCTGGATTCAT
Rat *Gli3*	CAAGGCTTTCTCTAACGCTT	GGGTCTGTGTAACGCTTGGT
Rat *Tnfα*	CCCAATCTGTGTCC	CACTACTTCAGCGTCT

	TTCTAA	CGTG
Murine *Epcam*	CCTCTGATGGACT GATTCTGCAC	ACACACGGACGAGACT GGAC
Murine *Sox9*	CACACGTCAAGCG ACCCATGAA	TCTTCTCGCTCTCGTT CAGCAG
Murine *Krt19*	ACCTACCTTGCTCG GATTG	TGACTTCGGTCTTGCT TAT
Murine *Krt7*	CGGAGATGAACCG CTCTATCCA	CATGAGCATCCTTGAT TGCCAGC
Murine *Col1a1*	CCTCAGGGTATTG CTGGACAAC	CAGAAGGACCTTGTTT GCCAGG
Murine *Col3*	GACCAAAAGGTGA TGCTGGACAG	CAAGACCTCGTGCTCC AGTTAG
Murine *Col4*	ATGGCTTGCCTGG AGAGATAGG	TGGTTGCCCTTTGAGT CCTGGA
Murine *Acta2*	AATGGCTCTGGGC TCTGTAA	TCTCTTGCTCTGGGCT TCAT
Murine *Tgf-β1*	AGTCAGAGACGTG GGGACTT	CGGAATAGGGGCGTCT GAG
Murine *Gli1*	CTCAAACTGCCCA GCTTAACCC	TGCGGCTGACTGTGTA AGCAGA
Murine *Smo*	AAGGCCACCCTGC TCATCTG	AGGCCTTGGCGATCAT CTTG
Murine *Dhh*	GCAGACCGCCTGA TGACAGA	GTTCATCACCGCGATG GCTA
Murine *Ptch2*	GGCACTCACATCC GTCAACAAC	GAAGACGAGCATTACC GCTGCA
Murine *Gli2*	ACACTGTGGAGGA CTGCCTACA	GGCATCTCCATGCCAC TGTCAT
Murine *Tnf-α*	GGTGCCTATGTCTC AGCCTCTT	GCCATAGAACTGATGA GAGGGAG
